# The Citrus Flavonoid Nobiletin Downregulates Angiopoietin-like Protein 3 (ANGPTL3) Expression and Exhibits Lipid-Modulating Effects in Hepatic Cells and Adult Zebrafish Models

**DOI:** 10.3390/ijms232012485

**Published:** 2022-10-18

**Authors:** Ching-Yen Lin, Pei-Yi Chen, Hao-Jen Hsu, Wan-Yun Gao, Ming-Jiuan Wu, Jui-Hung Yen

**Affiliations:** 1 Department of Molecular Biology and Human Genetics, Tzu Chi University, Hualien 970374, Taiwan; 2 Laboratory of Medical Genetics, Genetic Counseling Center, Hualien Tzu Chi Hospital, Buddhist Tzu Chi Medical Foundation, Hualien 970374, Taiwan; 3 Department of Life Science, Tzu Chi University, Hualien 970374, Taiwan; 4 Institute of Medical Sciences, Tzu Chi University, Hualien 970374, Taiwan; 5 Department of Biotechnology, Chia-Nan University of Pharmacy and Science, Tainan 717301, Taiwan

**Keywords:** nobiletin, ANGPTL3, lipoprotein lipase, TG-rich lipoprotein, LXRα

## Abstract

Nobiletin, a dietary citrus flavonoid, exerts biological activities against hyperlipidemia, obesity, and atherosclerotic cardiovascular diseases (ASCVDs). The aim of this study was to explore the lipid-lowering effects of nobiletin and the underlying molecular mechanisms in vitro in hepatic cells and in vivo in zebrafish models. Transcriptome and gene ontology (GO) analyses of differentially expressed genes (DEGs) by gene set enrichment analysis (GSEA) showed that a set of twenty-eight core enrichment DEGs associated with “GO BP regulation of lipid metabolic process” (GO: 0019216) were significantly downregulated in nobiletin-treated cells. Among these genes, angiopoietin-like 3 (ANGPTL3), an inhibitor of lipoprotein lipase (LPL) activity that regulates TG-rich lipoprotein (TGRL) metabolism in circulation, was the protein most markedly downregulated by nobiletin. Nobiletin (20 and 40 μM) significantly reduced the levels of ANGPTL3 mRNA and intracellular and secreted ANGPTL3 proteins in hepatic cell lines. Furthermore, alleviation of secreted ANGPTL3 production by nobiletin was found to reinstate LPL catalytic activity. Nobiletin significantly inhibited ANGPTL3 promoter activity and attenuated the transcription factor liver X receptor-α (LXRα)-mediated ANGPTL3 transcription. Molecular docking analysis predicted that nobiletin could bind to the ligand-binding domain of LXRα, thereby counteracting LXRα activation. In animal studies, orally administered nobiletin significantly alleviated the levels of plasma triglycerides (TGs) and cholesterol in zebrafish fed a high-fat diet. Moreover, nobiletin significantly reduced the amounts of hepatic ANGPTL3 protein in zebrafish. Our findings suggest that nobiletin may regulate the LXRα-ANGPTL3-LPL axis and exhibit lipid-modulating effects in vitro and in vivo. Thus, nobiletin is a potential ANGPTL3 inhibitor for the regulation of lipid metabolism to ameliorate dyslipidemia and ASCVDs.

## 1. Introduction

Dyslipidemia is a major causative factor in the development of atherosclerotic cardiovascular disorders (ASCVDs). It is characterized by dysregulation of lipid metabolic processes resulting in the excessive accumulation of low-density lipoprotein cholesterol (LDL-C), triglycerides (TG) and TG-rich lipoproteins (TGRLs) in plasma, and is highly associated with type 2 diabetes, obesity, metabolic syndrome and CVD [1]. High levels of plasma TGRL particles, including chylomicrons and VLDLs, are associated with dyslipidemia [2,3]. Lipolysis activation leads to reduced levels of atherogenic TGRLs, and increased clearance of cholesterol-rich lipoprotein remnants via hepatic cells is beneficial for patients with ASCVDs [4]. TG components in lipoproteins are hydrolyzed by lipoprotein lipase (LPL) in the capillary endothelium, resulting in the release of free fatty acids (FFAs) for utilization and oxidation by muscles, adipose tissues and the heart [5]. Recently, emerging evidence has shown that LPL overexpression or increases in LPL activity can reduce plasma TG content and increase high-density lipoprotein cholesterol (HDL-C) levels [6,7]; however, deficiency of LPL activity leads to hypertriglyceridemia [8]. These reports reveal that the restoration of LPL activity plays an important role in modulating plasma TG levels, lipid metabolism and homeostasis in the circulatory system.

LPL-mediated TGRL lipolysis plays an essential role in lipid metabolic processes, including lipid transportation, fatty acid metabolism and lipid accumulation for fuel or storage. Angiopoietin-like protein 3 (ANGPTL3), a member of the ANGPTL family that shares structural similarity to angiopoietin proteins, has been reported to be a critical inhibitor of LPL activity and to impede the hydrolysis of the TG portion in TGRLs in the capillary lumen [9,10,11]. ANGPTL3 is a secretory glycoprotein with a molecular weight of approximately 70 kDa that is mainly expressed and secreted by hepatocytes and can function in circulation, adipose tissue and muscle. The protein structure of ANGPTL3 contains several functional domains, including a signal peptide, specific epitope 1, an N-terminal coiled-coil domain, a linker region and a C-terminal fibrinogen-like domain [12,13,14]. The N-terminal coiled-coil region of ANGPTL3 is known to be a potent inhibitory domain for attenuation of LPL and endothelial lipase (EL) activity [15]. ANGPTL3 activity can be enhanced by ANGPTL8. ANGPTL3 alone or ANGPTL3/ANGPTL8 complexes can markedly inhibit LPL activity [16]. ANGPTL3 gene expression is mainly regulated by critical lipid metabolism-related transcription factors, such as liver X receptor α (LXRα) and hepatic nuclear factor 1α (HNF-1α), in hepatic cells. It has been reported that LXR is the most important regulator of ANGPTL3 expression [17,18]. The ANGPTL3 gene is a direct target of LXR, and overexpression of hepatic ANGPTL3 via activation of the LXR-dependent pathway leads to TG accumulation in the liver and hypertriglyceridemia [19]. These findings suggest that downregulation of LXR-dependent ANGPTL3 expression may be a novel strategy for the prevention or treatment of hypertriglyceridemia and ASCVDs.

Several studies have demonstrated that ANGPTL3 is an LPL suppressor and is responsible for the regulation of lipid metabolic processes in vivo. Genetic and clinical studies have revealed that deficiency, loss-of-function mutation, or downregulation of ANGPTL3 dramatically decreases the levels of TGs and LDL-C and the risk of lipid dysregulation and atherosclerotic cardiovascular events [20,21,22,23,24]. The conspicuous effect of ANGPTL3 on TGRL metabolism has sparked interest in ANGPTL3 as an alternative lipid-lowering target for the prevention or treatment of ASCVDs [25]. Recently, several studies performed in animal models and in human clinical trials for the management of dyslipidemia have focused on the development of pharmacological inhibitors of ANGPTL3, such as monoclonal antibodies for blocking ANGPTL3 activity and antisense oligonucleotides (ASOs) for reducing ANGPTL3 protein expression. These clinical studies indicated that inactivation of ANGPTL3 by inhibitors effectively ameliorates plasma TGs, LDL-C and lipoproteins in dyslipidemia, and thus, ANGPTL3 inhibition shows potential benefits in patients with ASCVDs [26,27,28].

The rational design of small molecules as ANGPTL3 inhibitors provides a potential approach for the management of dyslipidemia. Emerging evidence has shown that citrus polymethoxyflavones (PMFs) can modulate lipid metabolism to improve dyslipidemia, obesity and ASCVDs [29,30,31]. Nobiletin (5,6,7,8,3′,4′-hexamethoxyflavone) is a citrus PMF flavonoid that is mainly present in the peel of citrus fruits and has been reported to exhibit several biological or pharmacological activities, such as antioxidation, anti-inflammation, anticancer, anti-angiogenesis, anti-obesity, anti-diabetes, and neuroprotective effects [32,33,34,35,36,37]. Recently, in vitro and in vivo studies have focused on the bioactive function of nobiletin in the regulation of lipid metabolism. Nobiletin has been reported to inhibit lipid accumulation and exert antiadipogenic effects in 3T3-L1 cells [38]. Nobiletin inhibited apolipoprotein B secretion, cholesterol and TG synthesis, and lipogenesis and attenuated high glucose-induced lipid accumulation in HepG2 cells [39,40]. In animal studies, nobiletin alleviated adiposity and dyslipidemia by regulating the expression of lipid metabolism-related and adipokine genes in high-fat diet (HFD)-induced obese mice [41]. In HFD-fed rats, nobiletin increased the levels of adiponectin and superoxide dismutase and alleviated hyperlipidemia and nonalcoholic fatty liver disease (NAFLD) [42]. Nobiletin prevented obesity, hepatic steatosis, and dyslipidemia and ameliorated energy expenditure in mice fed a high fat/high cholesterol (HFHC) diet [43]. In HCD-fed mice, nobiletin reduced the LDL/VLDL to total cholesterol ratio, hepatic cholesterol and TG contents, and lipid accumulation by regulating lipid metabolism-related gene expression to ameliorate hypercholesterolemia and NAFLD [44]. In LDLR^−/−^ mice fed a HFHC diet, nobiletin prevented postprandial lipemia by attenuating TGRL levels in plasma and enhanced TGRL clearance through regulation of intestinal lipoprotein metabolism [45].

The aforementioned reports suggest that nobiletin possesses potential bioactivities to improve dysregulation of lipid homeostasis by modulating lipid and lipoprotein metabolic processes, but the underlying mechanisms remain to be elucidated. This study aimed to investigate the lipid-lowering effect of nobiletin, especially focusing on the modulation of ANGPTL3 gene expression, and to explore the underlying molecular mechanisms in hepatic cells and adult zebrafish.

## 2. Results

### 2.1. The Cytotoxic Effect of Nobiletin in Hepatic Cell Lines

**Figure 1:**
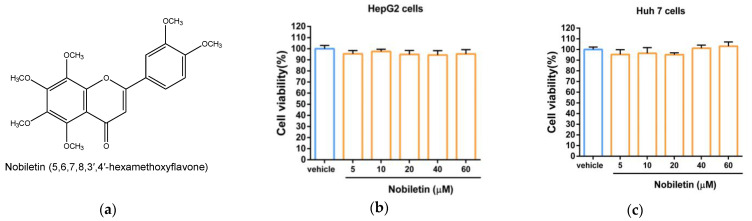
Effects of nobiletin on the viability of hepatic cells. (**a**) The chemical structure of nobiletin (5,6,7,8,3′,4′-hexamethoxyflavone). (**b**) HepG2 and (**c**) Huh 7 cells were treated with vehicle (0.1% DMSO) or nobiletin (5, 10, 20, 40 and 60 μM) for 24 h. Cell viability was analyzed with MTT assays. The data are shown as the mean ± SD of three independent experiments.

To examine the cytotoxic effect of nobiletin (Figure 1a) in hepatic cells, cells were incubated in medium containing vehicle (0.1% DMSO) or nobiletin (5–60 μM) for 24 h, and cell viability was analyzed using an MTT assay. As shown in Figure 1b, the data showed that no significant cytotoxicity of HepG2 cells was induced by nobiletin compared with vehicle-treated cells. Similar effects on cell viability were also demonstrated in nobiletin-treated Huh7 cells (Figure 1c).

### 2.2. Analysis of Nobiletin-Modulated Differentially Expressed genes (DEGs) in HepG2 Cells

**Figure 2:**
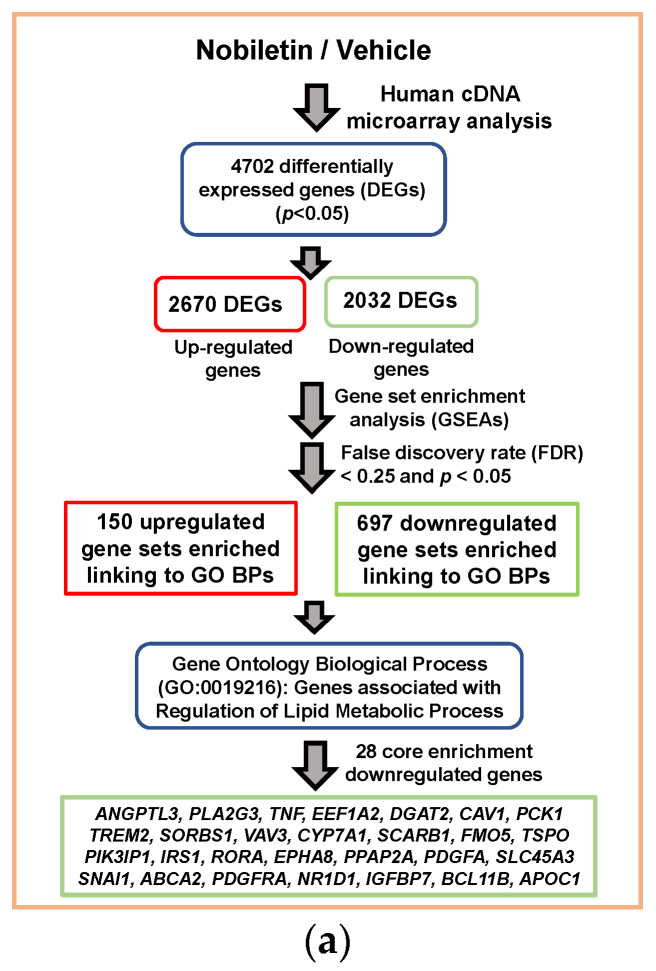

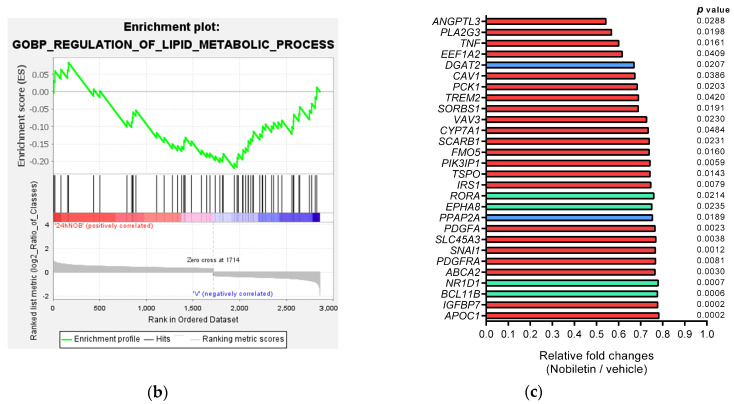
Microarray data and GSEA of DEGs for determination of gene ontology (GO) in response to nobiletin treatment in hepatic cells. HepG2 cells were treated with vehicle or nobiletin (40 μM) for 24 h, and the differential mRNA expression profiles were analyzed using a human cDNA microarray. (**a**) Flowchart of the transcriptome analysis and identification of DEGs in nobiletin-treated HepG2 cells. (**b**) GSEA demonstrating that the GO BP signature “Enrichment plot: GOBP Regulation of Lipid Metabolic Process” (GO: 0019216) gene set is enriched and downregulated with the DEGs in nobiletin-treated cells. The barcode shows the position of the genes involved in the gene set. (**c**) The fold changes of 28 significantly downregulated expression genes associated with GO:0019216 in response to nobiletin treatment (*p* < 0.05).

Nobiletin has been reported to exhibit lipid-modulating activities; therefore, the molecular changes involved in the modulation of lipid metabolism in response to nobiletin were elucidated via transcriptome analysis. To explore the potential genes involved in nobiletin-mediated lipid regulation, HepG2 cells were treated with vehicle or nobiletin (40 μM) for 24 h, and the differentially expressed gene (DEG) profiles were examined using a human genome-wide microarray system. In Figure 2a, a flowchart illustrating the transcriptome analysis result shows the DEGs in nobiletin-treated HepG2 cells. Microarray data showed that the selected significant 4702 DEGs were based on a *p* value less than 0.05. A total of 2670 upregulated and 2032 downregulated genes were identified in cells treated with nobiletin compared to the vehicle-treated cells. These DEGs were subjected to functional annotation with Gene Ontology (GO) enrichment using GSEA to determine the gene sets in which they are associated with biological processes (BPs). The data showed that 150 upregulated and 697 downregulated gene sets were enriched at a false discovery rate (FDR) < 0.25 and *p* < 0.05 and significantly linked to the respective GO BP terms (Tables S1 and S2). It was found that lipid metabolism related GOBPs, such as lipid localization, regulation of lipid transport, lipid storage, regulation of lipid metabolic process, lipid homeostasis, phospholipid transport, lipid catabolic process, neutral lipid metabolic process, and cellular lipid metabolic process were significantly changed by nobiletin.

Among these GOBPs, we focused on the “GOBP Regulation of Lipid Metabolic Process” (GO: 0019216) and analyzed the enrichment of transcriptional changes involved in this GO term via GSEA [Enrichment score (ES) = −0.220, normalized ES (NES) = −1.523, Nominal *p* value = 0.034, FDR *q* value = 0.034, leading edge: tags = 51%, list = 32%, signal = 73%]. The enrichment plot showed that twenty-eight core enrichment DEGs associated with GO:0019216 were markedly downregulated in nobiletin-treated cells (Figure 2b and Table 1). The transcriptional fold changes of the twenty-eight significantly downregulated genes in nobiletin-treated cells are shown in Figure 2c. Among these enriched genes, *ANGPTL3* was the most downregulated and considered an essential hub. ANGPTL3 has been reported to play a critical role in the inhibition of LPL activity to regulate atherogenic TGRL metabolism in plasma and dyslipidemia [11,46]. Therefore, the effect of nobiletin on ANGPTL3 expression was chosen for further investigation.

### 2.3. The Effect of Nobiletin on ANGPTL3 Gene Expression in Hepatic Cells

**Figure 3:**
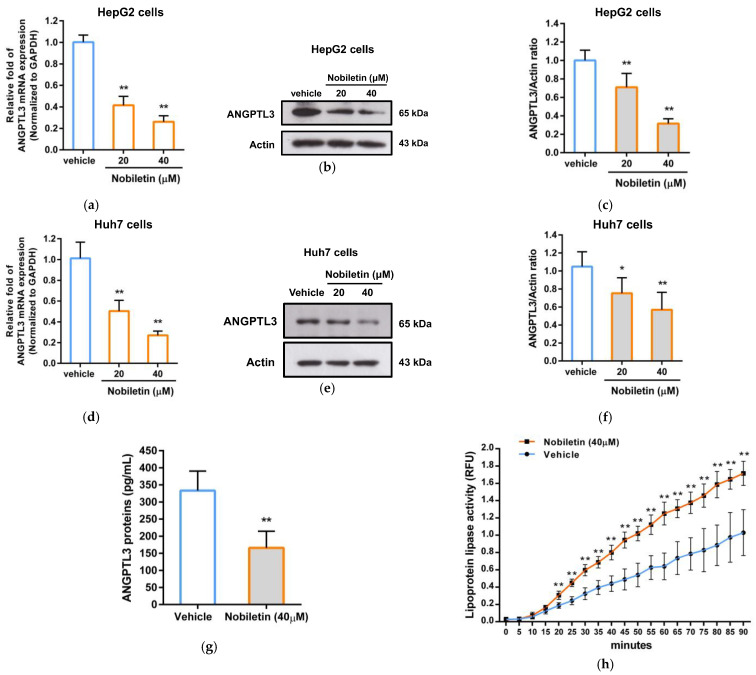
Effects of nobiletin on ANGPTL3 gene expression and LPL activity. HepG2 and Huh7 cells were treated with vehicle or nobiletin (20 and 40 μM) for 24 h. (**a**,**d**) The mRNA levels of ANGPTL3 and GAPDH were determined using RT–qPCR. (**b**,**e**) The protein levels of ANGPTL3 and actin were detected via Western blotting analysis. A representative blot is shown. (**c**,**f**) The normalized intensity of the signal for ANGPTL3 protein versus actin protein. (**g**) The culture medium from vehicle- or nobiletin (40 μM)-treated HepG2 cells was collected, and the extracellular ANGPTL3 protein was detected via ELISA. (**h**) The concentrated extracellular proteins (50 μg) from vehicle- or nobiletin (40 μM)-treated HepG2 cells and LPL enzyme were incubated with substrates to determine the catalytic activity of LPL via fluorogenic analysis. The fluorescence intensity was measured over 90 min. The data represent the mean ± SD of three independent experiments. * *p* < 0.05 and ** *p* < 0.01 indicate significant differences compared to the vehicle-treated group.

To verify whether nobiletin can regulate the gene expression of ANGPTL3, we examined the effect of nobiletin on the mRNA expression of ANGPTL3 using RT–qPCR analysis in hepatic cell lines. As shown in Figure 3a, nobiletin at 20 and 40 μM markedly decreased the mRNA level of ANGPTL3 by 0.40 ± 0.08- and 0.25 ± 0.06-fold in HepG2 cells, respectively, compared with the vehicle-treated cells (1.00 ± 0.07) (*p* < 0.01). Moreover, we investigated the ANGPTL3 protein level in nobiletin-treated cells via Western blot analysis. The cells treated with nobiletin showed significantly reduced levels of ANGPTL3 protein expression (0.76 ± 0.16- and 0.26 ± 0.11-fold) in HepG2 cells compared to the vehicle-treated cells (1.00 ± 0.11) (Figure 3b,c). Similar suppression of ANGPTL3 mRNA and protein expression by nobiletin were also found in Huh7 cells (Figure 3d–f). Furthermore, the levels of secreted extracellular ANGPTL3 protein in nobiletin-treated HepG2 cells were measured via ELISA. Figure 3g shows that nobiletin (40 μM) significantly decreased the levels of secreted ANGPTL3 protein in the medium by approximately 49.7%. These data suggest that nobiletin can significantly downregulate ANGPTL3 mRNA and protein expression in hepatic cells.

Moreover, we investigated whether the amount of secreted ANGPTL3 protein reduced by nobiletin can restore LPL activity. As shown in Figure 3h, LPL activity was significantly elevated by the culture supernatant collected from nobiletin-treated cells in a time-dependent manner. While incubation for 90 min, extracellular proteins of nobiletin-treated cells markedly increased LPL activity up to 1.7-fold compared to the vehicle-treated group. These data suggest that nobiletin downregulates ANGPTL3 gene expression and attenuates the production of secreted ANGPTL3 proteins in hepatic cells, leading to restoration of extrahepatic LPL activity.

### 2.4. Nobiletin Suppresses the Transcriptional Activity of the ANGPTL3 Promoter in HepG2 Cells

**Figure 4:**
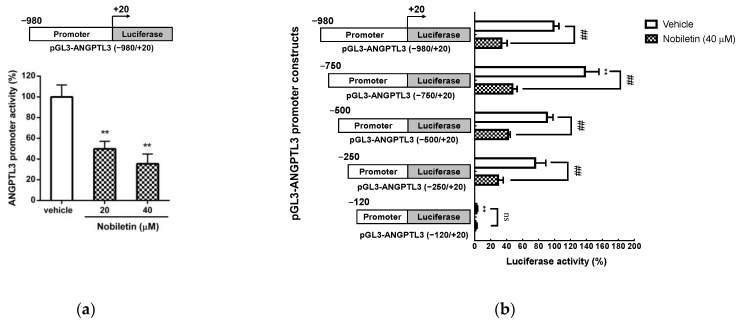
Effects of nobiletin on ANGPTL3 promoter activity. (**a**) HepG2 cells were co-transfected with the pGL3-ANGPTL3 (−980/+20) plasmid and *Renilla* control plasmid. After 24 h of transfection, the cells were treated with vehicle or nobiletin (20 and 40 μM) for 24 h. Luciferase activity was determined with a reporter assay kit. Data were normalized to *Renilla* control luciferase activity and represent the mean ± SD from three independent experiments. ** *p* < 0.01 indicates a statistically significant difference compared to the vehicle-treated group. (**b**) HepG2 cells were cotransfected with serially deleted ANGPTL3 promoter–luciferase reporter plasmids, including pGL3-ANGPTL3 (−750/+20), pGL3-ANGPTL3 (−500/+20), pGL3-ANGPTL3 (−250/+20), and pGL3-ANGPTL3 (−120/+20), and a control *Renilla* plasmid followed by treatment with vehicle or nobiletin (40 μM) for 24 h. Data represent the mean ± SD of three independent experiments. ** *p* < 0.01 indicates a significant difference compared to vehicle-treated pGL3-ANGPTL3 (−980/+20)-transfected cells. ## *p* < 0.01 indicates a significant difference compared to vehicle treatment of the respective deletion construct-transfected groups. “ns” indicates no significance.

According to the above results, nobiletin is able to inhibit the mRNA transcription of ANGPTL3; thus, we further investigated whether nobiletin can regulate the transcriptional activity of the ANGPTL3 promoter in hepatic cells. As shown in Figure 4a, the luciferase activity in the pGL3-ANGPTL3 (−980/+20)-transfected group was significantly reduced in nobiletin (20 and 40 μM)-treated cells compared to vehicle-treated cells (from 100.00 ± 11.59% to 46.60 ± 9.09% and 37.03 ± 10.09%, respectively) (*p* < 0.01). These data indicate that nobiletin significantly suppressed ANGPTL3 promoter activity in HepG2 cells. To further examine the mechanism underlying the nobiletin-mediated reduction in ANGPTL3 expression, the DNA elements within the ANGPTL3 promoter that mediate the response to nobiletin were analyzed. HepG2 cells were cotransfected with the *Renilla* control and the ANGPTL3 promoter constructs with a series of deletions, followed by treatment with vehicle or nobiletin (40 μM). As shown in Figure 4b, in cells transfected with the pGL3-ANGPTL3 (−750/+20), pGL3-ANGPTL3 (−500/+20) or pGL3-ANGPTL3 (−250/+20) constructs, nobiletin significantly reduced luciferase activity compared to that in vehicle-treated cells. However, the transcriptional activity was substantially reduced and no effect of nobiletin treatment was observed in cells transfected with pGL3-ANGPTL3 (−120/+20) plasmids. These results suggest that the DNA region between the −250 and −121 positions contains a critical responsive element for the regulation of hepatic ANGPTL3 expression by nobiletin.

### 2.5. Nobiletin Inhibits ANGPTL3 Expression by Counteracting LXRα Activity

**Figure 5:**
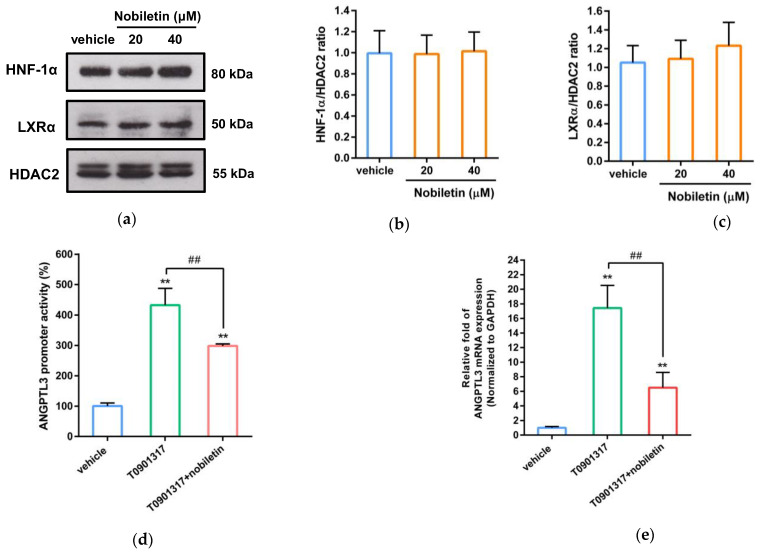
Effects of nobiletin on HFN-1α and LXRα protein expression and LXRα activity. HepG2 cells were treated with vehicle or nobiletin (20 and 40 μM) for 24 h. (**a**) The nuclear HFN-1α, LXRα and HDAC2 protein levels were detected by Western blot analysis. The normalized intensity of (**b**) HFN-1α and (**c**) LXRα versus HDAC2 is presented as the mean ± SD from three independent experiments. (**d**) HepG2 cells were cotransfected with a pGL3-ANGPTL3 p(−250/+20)) plasmid and a *Renilla* control plasmid for 24 h. The plasmid-transfected cells were pretreated with vehicle or nobiletin (40 μM) for 1 h followed by incubation with the LXRα agonist T0901317 (1 μM) for an additional 24 h. The luciferase activities were measured with a reporter assay kit and normalized to the respective *Renilla* luciferase activities. The data represent the mean ± SD from three independent experiments. (**e**) HepG2 cells were pretreated with vehicle or nobiletin (40 μM) for 1 h followed by treatment with T0901317 (1 μM) for an additional 24 h. ANGPTL3 mRNA was detected via RT–qPCR. Data are shown as the mean ± SD of three independent experiments. ** *p* < 0.01 indicates a significant difference compared to the vehicle-treated group. ## *p* < 0.01 indicates a significant difference compared to the group treated only with T0901317.

The nobiletin-responsive region in the ANGPTL3 promoter (from −250 to −121) contains two putative transcription factor-binding sites, liver X receptor α (LXRα, from −163 to −147 bp) and hepatocyte nuclear factor 1α (HNF-1α, from −136 to −122 bp), which have been reported to participate in the modulation of ANGPTL3 expression in hepatic cells [17]. Therefore, we further investigated whether nobiletin could regulate nuclear LXRα and HNF-1α proteins in HepG2 cells. As shown in Figure 5a–c, nobiletin did not change the protein levels of nuclear LXRα or HNF-1α in HepG2 cells. Therefore, we further investigated the effect of nobiletin on LXRα-mediated transcriptional activity. As shown in Figure 5d, ANGPTL3 promoter activity was significantly increased by the synthetic LXR agonist T0901317 in HepG2 cells. Moreover, LXRα-mediated transcriptional activation was markedly alleviated by cotreatment with nobiletin. As shown in Figure 5e, nobiletin significantly attenuated T0901317-induced mRNA expression of ANGPTL3 by approximately 63% (*p* < 0.01). These results showed that nobiletin can counteract LXRα-mediated ANGPTL3 mRNA transcription. These findings indicate that nobiletin downregulates ANGPTL3 expression via modulation of LXRα activity in hepatic cells.

### 2.6. Nobiletin Docks into the Ligand-Binding Domain (LBD) of LXRα

**Figure 6:**
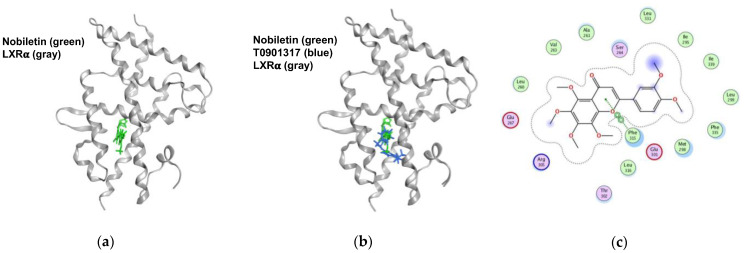
The poses of nobiletin docked into the ligand-binding domain (LBD) of LXRα. (**a**) The LXRα receptor is shown as a gray ribbon, and nobiletin is shown in green. (**b**) Superposition of both nobiletin and T0901317 (blue) bound to the LBD of LXRα. (**c**) Two-dimensional interaction map of LXRα and nobiletin. Nobiletin is surrounded by hydrophobic residues (green) and hydrophilic residues (pink) within the LBD of LXRα. Dotted line indicates a stacking interaction between the heterocyclic ring of nobiletin and the aromatic group of the phenylalanine (Phe315) residue.

To investigate whether nobiletin can interact with LXRα, molecular docking analysis was carried out as described in the Section 4. The docking results showed that nobiletin can bind to the LBD of LXRα, obtaining a docking score of −8.17 (Figure 6a). The superposition result showed that both nobiletin and T0901317 docked into the LXRα-LBD in similar poses (Figure 6b). As shown in Figure 6c, the ligand–receptor interaction map revealed that nobiletin was surrounded by several hydrophobic residues (Phe315, Leu316, Met298, Phe335, Ile295, Leu260, Leu331, Ala261 and Ile339) and some hydrophilic residues (Arg305, Glu301, Ser264 and Glu267) within the LBD of LXRα. The heterocyclic ring (C ring) of nobiletin is predicted to form a noncovalent bond with the aromatic ring of Phe315 via π-π stacking interactions. These data suggest that nobiletin has the potential to regulate the activity of LXRα via interaction with the hydrophobic pocket of LXRα-LBD.

### 2.7. Nobiletin Modulates the Levels of Plasma Lipids and Hepatic ANGPTL3 Expression in Adult Zebrafish

**Figure 7:**
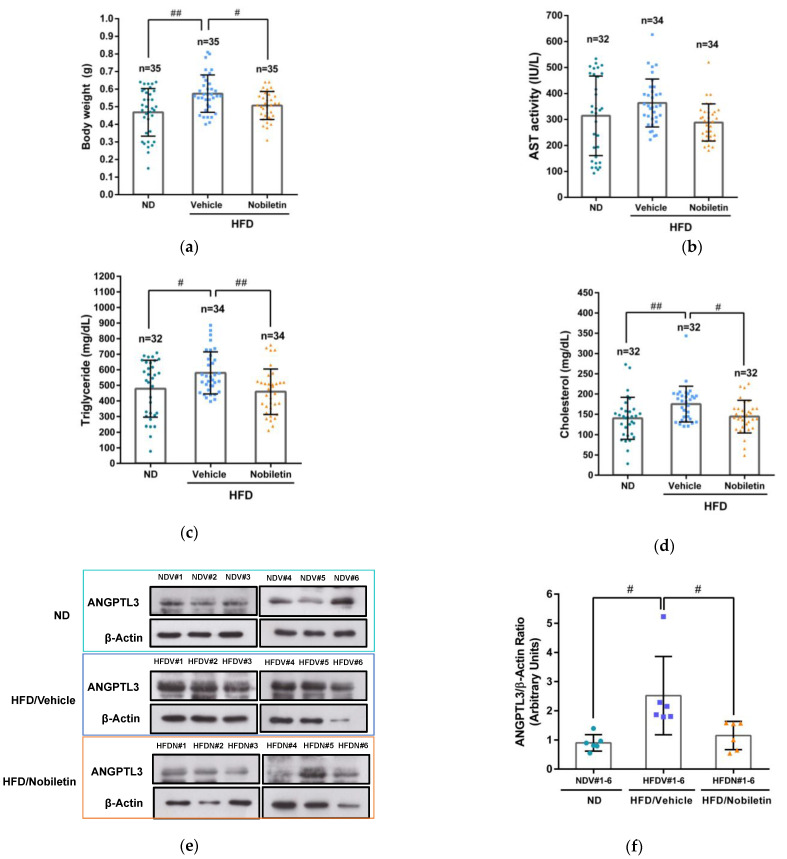

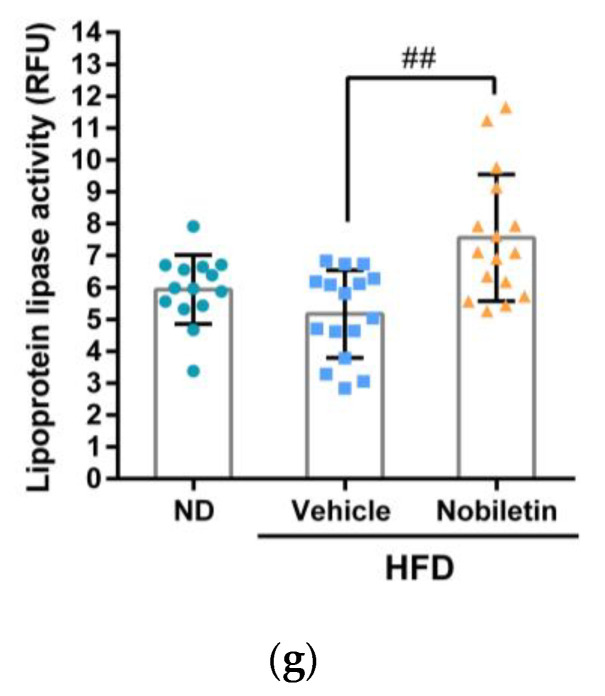
Nobiletin alleviated lipid levels and hepatic ANGPTL3 expression in adult zebrafish. Male adult zebrafish were allocated into three groups: normal diet plus vehicle control (ND), high-fat diet plus vehicle (HFDV) and high-fat diet plus nobiletin (HFDN). The zebrafish were administered vehicle or nobiletin (100 mg/kg) by oral gavage (one dose every two days) for two weeks. The (**a**) body weight, (**b**) AST activity, (**c**) triglyceride and (**d**) cholesterol contents were analyzed as described in Materials and Methods. Values are expressed as the means ± SD. # *p* < 0.05 and ## *p* < 0.01 represent significantly statistical differences compared to the ND- or vehicle-treated groups. (**e**) The protein extracts of livers from 6 individual zebrafish in respective ND, HFD/Vehicle, and HFD/Nobiletin groups (NDV#1~#6, HFDV#1~#6, and HFDN#1~#6) were prepared and the ANGPTL3 and β-actin proteins were detected by Western blotting analysis. A representative blot is shown. (**f**) The intensity of the protein signal for ANGPTL3 and β-actin was measured in different blots from replicate experiments. The normalized values of ANGPTL3 versus β-actin from 6 individual zebrafish in respective groups (NDV#1~#6, HFDV#1~#6, and HFDN#1~#6) are expressed as the means ± SD. # *p* < 0.05 represents significantly statistical differences compared to the HFD/Vehicle-treated groups. (**g**) The protein extracts of livers (10 μg) from zebrafish treated with ND (n = 14), HFD/Vehicle (n = 16) and HFD/Nobiletin (n = 16) groups and LPL enzyme were incubated with substrates to determine the LPL activity via fluorogenic analysis. The fluorescence intensity was measured at 90 min. The data represent the mean ± SD. ## *p* < 0.01 represents significantly statistical differences compared to the vehicle-treated groups.

To elucidate the lipid-modulating effects of nobiletin in vivo, adult male zebrafish were allocated into three groups including normal diet plus vehicle control (ND), high-fat diet plus vehicle (HFD/vehicle) and HFD plus nobiletin (HFD/nobiletin) groups for analysis of plasma lipids as described in Materials and Methods. In the HFD/vehicle-fed groups, the body weight, TG and cholesterol levels of zebrafish were significantly higher than those of the ND-treated group. In the HFD/nobiletin groups, the body weight of zebrafish was significantly lower than those of the HFD/vehicle-treated group (*p* < 0.01) (Figure 7a). The AST activity was slightly reduced in HFD/nobiletin-administered zebrafish as compared to the HFD/vehicle treatment groups (Figure 7b). As shown in Figure 7c,d, in the HFD-fed groups, the plasma TG and cholesterol levels in nobiletin-treated zebrafish were markedly alleviated by approximately 21% and 16%, respectively, compared with levels in vehicle-treated fish. These data indicate that nobiletin can ameliorate high amounts of TG and cholesterol in the circulation. Furthermore, we determined whether nobiletin can modulate the protein level of hepatic ANGPTL3 in zebrafish. As shown in Figure 7e,f, in the HFD-fed groups, the hepatic ANGPTL3 protein level was lower in the nobiletin-treated zebrafish than those of the vehicle treatment groups. Moreover, we examined the effect of nobiletin-mediated hepatic ANGPTL3 reduction in zebrafish on LPL activity. As shown in Figure 7g, in the HFD-fed groups, LPL activity was significantly increased by the liver lysates from nobiletin-administered zebrafish compared to the vehicle group. These results suggested that nobiletin reduces hepatic ANGPTL3 expression, concomitant with the modulation of LPL activity and lipid metabolism to remedy dyslipidemia in vivo.

## 3. Discussion

**Figure 8:**
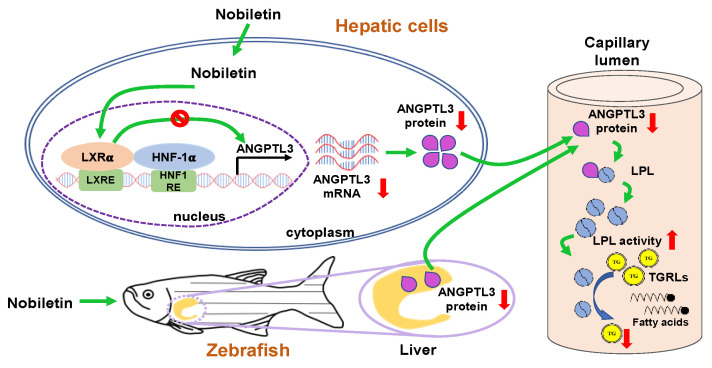
A hypothetical mechanism by which nobiletin regulates lipid metabolism through modulation of the LXRα-ANGPTL3-LPL axis in vitro and in vivo. In vitro, nobiletin downregulates the mRNA and protein expression of ANGPTL3 in hepatic cell lines by counteracting the activation of LXRα, thereby reducing ANGPTL3 secretion and leading to increased LPL activity in the capillary lumen. In vivo, hepatic ANGPTL3 protein levels are alleviated in HFD-fed, nobiletin-treated zebrafish and potentially enhance the clearance of TGRLs and reduce TG accumulation in circulation.

Mounting evidence supports citrus flavonoids possessing a potent activity to modulate lipid metabolic processes against dyslipidemia and atherosclerosis. Herein, this is the first report to show that nobiletin significantly downregulated ANGPTL3 mRNA and protein expression through counteraction of the transcriptional activity of LXRα in hepatic cells. The reduction in secreted ANGPTL3 proteins by nobiletin may enhance LPL activity, resulting in a reduction in TG accumulation in the bloodstream. Furthermore, nobiletin also reduced hepatic ANGPTL3 expression and alleviated the amounts of plasma TG and cholesterol in HFD fed zebrafish. These findings reveal that nobiletin has a potential to modulate TGRL and lipid metabolism in circulation through downregulation of ANGPTL3 expression in hepatic cells (Figure 8).

Nobiletin is one of the abundant PMFs existing in citrus peels and has been shown to possess biological abilities for modulation of lipid metabolism and homeostasis. Several reports suggested that the range of PMFs dosage was used from 1 to 100 μM in in vitro studies and 10 to 200 mg/kg in selected animal models, respectively [31]. In this study, nobiletin (5–60 μM) has no cytotoxicity and is effective at the concentration of 20 and 40 μM in hepatic cell lines. Orally administered nobiletin (100 mg/kg) exhibited significant lipid-lowering effects in HFD zebrafish. These results confirm that citrus nobiletin is a safe flavonoid and may serve as a nutraceutical ingredient in dietary supplements. However, the poor solubility and low oral bioavailability of citrus nobiletin lead to limiting their effectiveness in vivo. Therefore, to increase the oral bioavailability of nobiletin in vivo, chemical modification and novel encapsulated or nano-emulsifying delivery systems may improve its solubility and stability to optimize effective dosage in animal and clinical studies [47,48,49,50]. Furthermore, establishing nobiletin delivery methods for clinical application requires more pharmacokinetic and pharmacodynamic studies in the future.

For evaluation in the early steps of new drug discovery, transcriptomic analysis is a valuable strategy to explore functional molecules and pathways that respond to compounds [51]. In this study, transcriptome data elucidated the molecular mechanisms by which hepatic cells respond to nobiletin, leading to modulation of lipid metabolism. Using GSEA and GO enrichment analyses of transcriptomic data, nobiletin was found to significantly downregulate the expression of a gene set linked to the GO BP regulation of lipid metabolic process (GO: 0019216), which defines genes involved in any process that modulates the chemical reactions and pathways involved in lipid metabolism. Twenty-eight core enrichment DEGs associated with GO:0019216 were markedly downregulated by nobiletin treatment. Among these genes, the *ANGPTL3* gene was discovered to have the greatest decrease in mRNA expression in nobiletin-treated HepG2 cells. ANGPTL3 is a glycoprotein secreted by hepatocytes that suppresses the catalytic activity of lipases, including lipoprotein and hepatic lipases. Among these DEGs, we also found that nobiletin downregulated the expression of *SCARB1*, a scavenger receptor class B type I (SR-BI) glycoprotein highly expressed in hepatocytes. The hepatic SR-BI protein plays a critical role in regulating HDL-C metabolism and modulating lipoprotein homeostasis. SR-BI on the hepatic cell membrane binds to HDL particles and mediates the selective uptake of cholesterol esters (CEs) in HDL as well as the reverse cholesterol transport (RCT) process [52]. In human studies, *SCARB1* gene mutations that reduce the protein and impede the ability of the liver to uptake CEs in HDL particles, result in elevated plasma HDL-C levels and the risk of CVDs [53]. Recently, in vitro and in vivo studies reported that SR-BI-independent mechanisms were also involved in HDL selective CEs uptake by the liver [54,55]. In this study, nobiletin slightly decreased the mRNA level of SR-BI by 0.75-fold in HepG2 cells as compared with the vehicle-treated cells. Whether inhibition of hepatic SR-BI expression by nobiletin leads to increase plasma HDL-C levels remains unclear and needs to be further investigated.

Clinical studies have shown that inactivation or downregulation of ANGPTL3 using high-cost monoclonal antibodies (such as Evinacumab) or antisense oligonucleotides (such as Vupanorsen) dramatically alleviates plasma LDL-C and triglyceride levels [20,56]. However, the absence of oral ANGPTL3 inhibitors has limited the therapeutic effect of these novel medications. To date, few small molecules have been studied and reported to function as ANGPTL3 inhibitors. Recently, emerging evidence has suggested that identifying compounds from natural phytochemicals that target ANGPTL3 is a feasible strategy for the prevention or treatment of dyslipidemia and ASCVDs. Xiao et al. reported that 1,3,5,8-tetrahydroxyxanthone, a natural flavonoid, reduced plasma ketone bodies, triglycerides, total cholesterol levels and hepatic ANGPTL3 expression concomitantly with increased adipose LPL expression in ketosis mice [57]. Our previous in vitro study demonstrated that tangeretin suppressed the mRNA and protein expression levels of ANGPTL3 in hepatic cells, resulting in increases in LPL activity [58]. In the present study, nobiletin (20–40 μM), a citrus flavonoid phytochemical, significantly decreased the ANGPTL3 mRNA and protein levels in HepG2 and Huh7 hepatic cell lines. The production of secreted ANGPTL3 proteins was also alleviated by nobiletin treatment. The enzymatic activity of LPL was recovered upon incubation with extracellular proteins in medium collected from nobiletin-treated cells. In the HFD zebrafish models, nobiletin markedly attenuated ANGPTL3 protein levels in the liver and lessened the amounts of TG and cholesterol in plasma. These data indicate that nobiletin downregulates ANGPTL3 gene expression in hepatocytes and diminishes the secreted form of ANGPTL3 concomitantly with the restoration of LPL activity in blood capillaries, resulting in alleviation of lipid levels in the circulation. Our findings show that nobiletin can modulate ANGPTL3 expression in vitro and in vivo and may serve as a preventive or therapeutic agent for lipid management.

LXRs have been reported to regulate the expression of genes involved in several lipid metabolic processes, including lipid and cholesterol absorption, lipogenesis and lipoprotein production and transportation. Modulation of the LXR pathway might provide a novel therapeutic strategy against dysregulation of lipid metabolism [59]. Liver-secreted ANGPTL3 is known to be mainly stimulated by LXRα activation [60]. In this study, the amount of nuclear LXRα protein was not changed by nobiletin. However, nobiletin significantly abrogated the LXRα agonist T0901317-induced ANGPTL3 promoter activation as well as mRNA expression in hepatic cells. The LBD (amino acid residues 206 to 447) of LXRα is known to fold into a hydrophobic pocket suitable for interaction with lipophilic compartments of compounds. In this study, molecular docking data predicted that nobiletin interacts with the LBD of LXRα. The interaction map showed that hydrophobic amino acid residues within the T0901317 binding pocket seem to be preferentially attracted by the methoxy groups in nobiletin. The Phe315 of LXRα was predicted to form a stacking interaction with the heterocyclic ring structure of the flavonoid nobiletin. These data indicate that nobiletin may specifically bind to the ligand-binding pocket of LXRα and thereby alleviate LXRα-mediated ANGPTL3 expression. Choi et al. reported that nobiletin inhibited adipogenesis by activation of AMPK-dependent pathway in 3T3-L1 cells [38]. Yuk et al. reported reduced SREBP-1c and FASN expression through activation of the AMPK pathway and attenuated high glucose-induced lipid accumulation in HepG2 cells [40]. AMPK is known to play a critical role in regulation of lipogenesis and lipid metabolism in hepatocytes [61]. AMPK also has been reported to play a role in inhibiting LXRα-mediated SREBP-1c transcription in HepG2 cells [62]. In this study, nobiletin could inhibit LXRα agonist induced ANGPTL3 expression in HepG2 cells. Whether the inhibition of the LXRα/ANGPTL3 pathway by nobiletin is also dependent on AMPK activation remains to be further investigated.

In this study, we used the HFD-fed adult zebrafish models to investigate the lipid-lowering effect of nobiletin in vivo. Emerging evidence supports the notion that zebrafish are useful for studying lipid metabolic disorders and hypolipidemic drugs because glucose and lipid metabolic pathways as well as fat storage mechanisms are conserved between fish and mammals [63,64]. Ka et al. reported an overall similarity in pathways regulated in zebrafish and mice upon an HFD regimen, which indicates that zebrafish are an emerging model for investigation of the pathogenesis or treatment of dyslipidemia and associated diseases in humans [65]. In this study, we found that nobiletin ameliorated plasma TG and cholesterol levels in HFD-fed zebrafish. We further demonstrated that the level of ANGPTL3 protein in the liver was reduced by nobiletin in zebrafish. ANGPTL3 was reported to be produced in the liver and regulates lipid metabolism in zebrafish. Zebrafish with downregulation of ANGPTL3 expression showed the hypocholesterolemia phenotype [66]. These findings support the idea that nobiletin can modulate hepatic ANGPTL3 expression and exert TG- and cholesterol-lowering effects in vivo.

In conclusion, our study demonstrated that nobiletin is a powerful ANGPTL3 inhibitor. Nobiletin can regulate lipoprotein metabolism and plasma lipid homeostasis by modulating the LXRα-ANGPTL3-LPL axis. Our findings suggest that nobiletin may serve as a potentially preventive or therapeutic agent for dyslipidemia and ASCVDs through downregulation of ANGPTL3 expression. In this study, we demonstrated the lipid-modulating effects of nobiletin in vitro in cell lines and in vivo in zebrafish. Further consideration and investigation in human studies focusing on safe doses, efficacy and bioavailability are required to carry forward the use of nobiletin into the nutraceutical or clinical arena in the future.

## 4. Materials and Methods

### 4.1. Chemicals

Nobiletin was purchased from Enzo Life Sciences (Farmingdale, New York, NY, USA). T0901317, 3-(4,5-dimethyl-2-thiazolyl)-2,5-diphenyl-2H-tetrazolium bromide (MTT) and dimethyl sulfoxide (DMSO) were purchased from Sigma–Aldrich Co. (Saint Louis, MO, USA). DMEM, fetal bovine serum (FBS) and nonessential amino acids (NEAAs) were purchased from Thermo Fisher Scientific, Inc. (Waltham, MA, USA).

### 4.2. Cell Culture and Compounds Treatment

HepG2 and Huh7 cells were obtained and cultured as previously described [58]. HepG2 cells were purchased from the Bioresource Collection and Research Center (Hsinchu, Taiwan). Huh7 cells were kindly provided by Professor Ming-Fu Chang (National Taiwan University, Taipei, Taiwan). For treatment of nobiletin, cells were treated with vehicle (0.1% DMSO) or various concentrations of nobiletin for 24 h. For treatment of the LXRα agonist T0901317, cells were pretreated with nobiletin (40 μM) for 1 h, followed by incubation with T0901317 (1 μM) for an additional 24 h.

### 4.3. Measurement of Cell Viability

The cells were treated with vehicle or nobiletin (5–60 μM) for 24 h. MTT reagent (1 mg/mL) was added to each well and incubated with cells at 37 °C for 3 h. After incubation, the medium was removed, and the purple formazan crystals were dissolved in DMSO. The viability of cells was determined by measuring the absorbance at 550 nm using a microplate reader. The percentage of cell viability in each group was calculated as follows: (OD_550_ of compound treatment/OD_550_ of vehicle control) × 100%.

### 4.4. RNA Preparation and Transcriptome Analysis

Total cellular RNA was prepared for transcriptome analysis using the human cDNA microarray as previously described [67]. Briefly, RNA was isolated from vehicle- or nobiletin (40 μM)-treated HepG2 cells using a Cytiva illustra^TM^ RNASpin Mini Isolation Kit (Cytiva, Marlborough, MA, USA) according to the manufacturer’s instructions. Fluorescence targets were prepared and hybridized using Human Whole Genome One Array Plus Version 7.1 (HOA 7.1, Phalanx Biotech Group, Hsinchu, Taiwan). The signals were scanned, and the data were analyzed with GenePix 4.1 software (Molecular Devices, Sunnyvale, CA, USA) and a Rosetta Resolver 7.2 System (Rosetta Biosoftware, Seattle, WA, USA). The intensities of each spot were normalized and transformed to the log_2_[fold change] of gene expression. Differentially expressed genes (DEGs) in response to nobiletin treatment with a *p* value < 0.05 were selected for further analysis.

### 4.5. Gene Ontology (GO) and Gene Set Enrichment Analysis (GSEA)

Gene Ontology (GO) term enrichment of significant DEGs (*p* < 0.05) from the human cDNA microarray was analyzed via Gene Set Enrichment Analysis (GSEA) to determine the gene sets linked to GO biological processes (BPs). GSEA using the Molecular Signatures Database (MSigDB) (http://software.broadinstitute.org/gsea/(accessed on 13 June 2022)) was performed to explore the statistically significant GO BPs which were involved in the response of cells to nobiletin treatment [58,68].

### 4.6. Reverse-Transcription Quantitative PCR (RT–qPCR) Analysis

The hepatic cell lines were treated with vehicle or nobiletin (20 and 40 μM) for 24 h. After treatment, the cells were harvested, and total cellular RNA was extracted using a blood/cultured cell total RNA purification mini kit (FAVORGEN Biotech, Ping-Tung, Taiwan) according to the manufacturer’s instructions. The isolated RNA was reverse transcribed into cDNA using a High-Capacity cDNA Reverse Transcription kit (Thermo Fisher Scientific). Quantitative real-time PCR was performed using a reaction mixture containing cDNA, human-specific primers (ANGPTL3, forward: 5′-tcctgctgaatgtaccacca-3′ and reverse: 5′-tcttctctaggcccaaccaa-3′; GAPDH, forward: 5′-catgagaagtatgacaacagcct-3′ and reverse: 5′-agtccttccacgataccaaagt-3′) [58] and Maxima SYBR Green/Rox qPCR Master Mix (Thermo Fisher Scientific). PCR amplification was performed with a Roche LightCycler^®^-480 Real-Time PCR System (Roche Diagnostics, Rotkreuz, Switzerland) according to the manufacturer’s instructions. The experimental mRNA levels of each target gene were normalized to that of the control GAPDH mRNA in the same sample. The relative differences in mRNA expression were calculated using the ∆∆C_t_ method.

### 4.7. Western Blot Analysis

Cells were harvested, and total cellular protein was extracted using RIPA buffer (Thermo Fisher Scientific). Nuclear proteins were prepared using a Nuclear Extract Kit (Active Motif, Carlsbad, CA, USA). The proteins from each sample were separated via 10% SDS–PAGE and transferred to PVDF membranes (Cytiva). The membranes were incubated with the following specific primary antibodies: anti-ANGPTL3 (Cat#A5225, RRID:AB_2863493, for the human cell line samples) (ABclonal, Woburn, MA, USA), anti-ANGPTL3 (Cat#PA5-72812, RRID:AB_2718666, for the zebrafish samples) (Thermo Fisher Scientific), anti-LXRα (Cat#ab41902, RRID:AB_776094) (Abcam, Cambridge, UK), anti-HDAC2 (Cat#GTX112957, RRID:AB_1950480) (GeneTex, Irvine, CA, USA), anti-Actin (Cat#MAB1501, RRID:AB_2223041) (Millipore Sigma-Aldrich) and anti-HNF-1α (Cat#89670, RRID:AB_2728751), anti-β-actin (Cat#8457, RRID:AB_10950489) (Cell Signaling Technology, Danvers, MA, USA). After that, the membranes were incubated with the appropriate horseradish peroxidase (HRP)-conjugated goat anti-rabbit (Cat#GTX213110-01, RRID:AB_10618573) (GeneTex) or anti-mouse (Cat#7076, RRID:AB_330924) (Cell Signaling Technology) IgG secondary antibodies. The membranes were rinsed with Amersham ECL^TM^ Prime Western blotting detection reagent, and the chemiluminescent signal bands were visualized with Amersham Hyperfilm^TM^ ECL (Cytiva).

### 4.8. Measurement of Extracellular ANGPTL3 Proteins

The amounts of extracellular ANGPTL3 proteins in the culture medium were measured via ELISA as previously described [58]. HepG2 cells were treated with vehicle or nobiletin (40 μM) for 24 h. The ANGPTL3 protein in culture supernatants of vehicle- or nobiletin-treated cells was collected and measured using a RayBio^®^ Human ANGPTL3 ELISA Kit (RayBiotech, Norcross, GA, USA) according to the manufacturer’s instructions. The absorbance was determined at 450 nm.

### 4.9. Analysis of Lipoprotein Lipase (LPL) Activity

Cells were cultured in DMEM (10% FBS and 1% NEAA) for 24 h. The medium was replaced with serum-free DMEM containing vehicle or nobiletin (40 μM), and the cells were incubated for an additional 24 h. After treatment, the conditioned medium of each sample was collected and concentrated using a Vivaspin^®^ 20 centrifugal concentrator (Sartorius, Göttingen, Germany). Lipase activity was detected using a Lipoprotein Lipase (LPL) Activity Assay Kit (Fluorometric) (Cell Biolabs, San Diego, CA, USA) according to the manufacturer’s instructions. Briefly, 50 μg of protein sample was mixed with 15.625 mUnits/mL LPL enzyme and LPL fluorometric substrate, and then, the mixtures were incubated at 37 °C and the fluorescence (485 nm excitation/525 nm emission) was read every 5 min for 90 min in a Varioskan^TM^ LUX multimode microplate reader (Thermo Fisher Scientific).

### 4.10. Plasmid Transfection and Measurement of ANGPTL3 Promoter Activity

The ANGPTL3 promoter–luciferase reporter plasmids pGL3-ANGPTL3 (−980/+20), pGL3-ANGPTL3 (−750/+20), pGL3-ANGPTL3 (−500/+20), pGL3-ANGPTL3 (−250/+20), and pGL3-ANGPTL3 (−120/+20) were generated as previously described [58]. HepG2 cells were cotransfected with these ANGPTL3 promoter-reporter plasmids and a pRL *Renilla* Luciferase Control Reporter Vector (Promega, Madison, WI, USA) using Lipofectamine 2000 Reagent (Thermo Fisher Scientific). After 24 h of transfection, vehicle or nobiletin (20 or 40 μM) was added and incubated with the cells for an additional 24 h. Luciferase activity was measured using a Dual-Luciferase Reporter Assay System Kit (Promega) and normalized to *Renilla* luciferase activity.

### 4.11. Molecular Docking of Nobiletin to the LXRα Protein

Molecular docking analysis was performed as previously described [69]. Briefly, to enhance the precision of the preferable binding sites between the LXRα receptor and compounds (nobiletin or T0901317), the “Induced fit” refinement within the DOCK module of the Molecular Operating Environment (MOE2019.01) software program was used to perform the molecular docking studies. Nobiletin and T0901317 were manually constructed using the MOE software package to dock with the LXRα binding domain (PDB: 3IPQ, LXRα with compound GW3965). Before docking, the crystal water molecules were eliminated, the missing short loops were added using MOE software, and the energy was minimized. GBVI/WSA is a force field-based scoring function for measuring ligand–receptor binding free energy. The binding free energy was the lowest S value of the scoring function. The lowest binding free energy determines the preferred binding site for each compound.

### 4.12. Zebrafish Experiments and Compound Administration

In zebrafish experiments, adult male zebrafish of the wild-type AB line were used. Zebrafish were purchased from Azoo Bio Corporation (Taipei, Taiwan) and maintained in a 14 h light/10 h dark cycle at 28 °C at the zebrafish facility in the Laboratory Animal Center of Tzu Chi University. All animal experiments were approved by the Institutional Animal Care and Use Committee of Tzu Chi University.

Zebrafish were allocated into two dietary groups: one group was fed a normal diet (ND, pellet food, Zeigler Brothers, Gardners, PA, USA), and the other group was fed a high-fat diet (HFD) (normal diet plus 20% (*w*/*w*) lard oil (Sigma)). Each zebrafish was administered 16.5 mg of the ND or HFD per day for 6 weeks. At the end of week 4, the HFD-fed group was subdivided into vehicle- and nobiletin-treated groups. ND group was administered vehicle (DMSO) and HFD-fed groups were administered vehicle or nobiletin (100 mg/kg of bodyweight) by oral gavage once every two days for 14 days. At the endpoint, zebrafish were fasted overnight before sampling. Zebrafish were euthanized, and body mass and length were measured. Blood samples were harvested using a tail ablation method as previously described [70]. Plasma was immediately collected and stored at −80 °C until analysis of blood lipids. Subsequently, liver samples were carefully resected, immediately transferred to a microcentrifuge tube and frozen at −80 °C. For hepatic protein preparation, zebrafish liver samples were homogenized and extracted using RIPA buffer for further analysis of protein expression.

### 4.13. Biochemical Analysis of Plasma Lipids in Zebrafish

The TG and cholesterol contents and aspartate aminotransferase (AST) activity in zebrafish blood samples were measured using a Triglyceride Colorimetric Assay Kit (Cayman Chemical, Ann Arbor, MI, USA), Cholesterol CHOD PAP Kit (Fortress Diagnostics, Antrim, UK) and Aspartate Aminotransferase (AST/GOT) Activity Assay Kit (Elabscience, Houston, TX, USA), respectively, according to the manufacturer’s instructions.

### 4.14. Statistical Analysis

For all experiments, at least three independent experiments were performed. The data are presented as the mean ± SD. Graph Pad Prism (version 9) (Graph Pad Software Inc., San Diego, CA, USA), was used to calculate *p* values via a Student’s *t*-test (two groups) or one-way ANOVA (multiple groups) with Tukey’s method for multiple comparisons. *p* values less than 0.05 represent “statistically significant” differences.

## Figures and Tables

**Table 1:** The twenty-eight core enrichment genes associated with GO BP regulation of lipid metabolic process (GO:0019216) were downregulated by nobiletin treatment.

Gene Symbol	Description	Rank in Metric Score	Running ES	log_2_(Nobiletin/Vehicle)
*ANGPTL3*	angiopoietin like 3	−0.890	0.012	−0.883
*PLA2G3*	phospholipase A2 group III	−0.826	−0.016	−0.817
*TNF*	tumor necrosis factor	−0.739	−0.034	−0.738
*EEF1A2*	eukaryotic translation elongation factor 1 alpha 2	−0.699	−0.054	−0.700
*DGAT2*	diacylglycerol O-acyltransferase 2	−0.591	−0.044	−0.577
*CAV1*	caveolin 1	−0.578	−0.063	−0.570
*PCK1*	phosphoenolpyruvate carboxy kinase 1	−0.547	−0.066	−0.547
*TREM2*	triggering receptor expressed on myeloid cells 2	−0.545	−0.085	−0.535
*SORBS1*	sorbin and SH3 domain containing 1	−0.538	−0.101	−0.537
*VAV3*	vav guanine nucleotide exchange factor 3	−0.478	−0.083	−0.462
*CYP7A1*	cytochrome P450 family 7 subfamily A member 1	−0.462	−0.088	−0.447
*SCARB1*	scavenger receptor class B member 1	−0.458	−0.089	−0.436
*FMO5*	flavin containing dimethylaniline monoxygenase 5	−0.442	−0.099	−0.439
*TSPO*	translocator protein	−0.432	−0.094	−0.431
*PIK3IP1*	phosphoinositide-3-kinase interacting protein 1	−0.432	−0.115	−0.431
*IRS1*	insulin receptor substrate 1	−0.425	−0.100	−0.424
*RORA*	RAR related orphan receptor A	−0.410	−0.122	−0.398
*EPHA8*	EPH receptor A8	−0.407	−0.114	−0.414
*PPAP2A*	phosphatidic acid phosphatase type 2A	−0.402	−0.127	−0.409
*PDGFA*	platelet derived growth factor subunit A	−0.396	−0.135	−0.388
*SLC45A3*	solute carrier family 45 member 3	−0.395	−0.142	−0.380
*SNAI1*	snail family transcriptional repressor 1	−0.389	−0.156	−0.384
*ABCA2*	ATP binding cassette subfamily A member 2	−0.383	−0.162	−0.389
*PDGFRA*	platelet derived growth factor receptor alpha	−0.383	−0.180	−0.383
*NR1D1*	nuclear receptor subfamily 1 group D member 1	−0.371	−0.167	−0.359
*IGFBP7*	insulin like growth factor binding protein 7	−0.368	−0.179	−0.364
*BCL11B*	BAF chromatin remodeling complex subunit BCL11B	−0.368	−0.203	−0.366
*APOC1*	apolipoprotein C1	−0.361	−0.189	−0.354

## Data Availability

Not applicable.

## Supplementary Materials

The following supporting information can be downloaded at: https://www.mdpi.com/article/10.3390/ijms232012485/s1.
