# Supplementary material for: The Citrus Flavonoid Nobiletin Downregulates Angiopoietin-like Protein 3 (ANGPTL3) Expression and Exhibits Lipid-Modulating Effects in Hepatic Cells and Adult Zebrafish Models
Source: Int J Mol Sci. 2022 Oct 18;23(20):12485. doi: 10.3390/ijms232012485 (PMC9604320; doi:10.3390/ijms232012485)
Supplement: Supplementary file 1 [file ijms-23-12485-s001.zip › ijms-1953817-supplementary.pdf]

Table S1. Biological processes in the Gene Ontology (GO) analysis of upregulated gene sets in nobiletin-treated HepG2 cells.

Gene Sets Follow link to MsigDB	Enrichment Score (ES)	Nominal ES (NES)
GOBP_RIBONUCLEOPROTEIN_COMPLEX_BIOGENESIS	0.43	3.48
GOBP_NCRNA_PROCESSING	0.41	3.35
GOBP_NCRNA_METABOLIC_PROCESS	0.38	3.31
GOBP_RIBOSOME_BIOGENESIS	0.42	3.22
GOBP_RNA_PROCESSING	0.32	3.06
GOBP_RRNA_METABOLIC_PROCESS	0.4	2.81
GOBP_DNA_REPAIR	0.32	2.67
GOBP_DNA_REPLICATION	0.34	2.52
GOBP_ENDOPLASMIC_RETICULUM_ORGANIZATION	0.54	2.48
GOBP_TRNA_METABOLIC_PROCESS	0.39	2.47
GOBP_MRNA_METABOLIC_PROCESS	0.28	2.46
GOBP_MITOCHONDRIAL_GENE_EXPRESSION	0.42	2.41
GOBP_CHROMOSOME_SEGREGATION	0.32	2.4
GOBP_DOUBLE_STRAND_BREAK_REPAIR	0.34	2.38
GOBP_MRNA_PROCESSING	0.31	2.37
GOBP_GOLGI_VESICLE_TRANSPORT	0.36	2.36
GOBP_TRANSLATIONAL_INITIATION	0.46	2.36
GOBP_ENDOPLASMIC_RETICULUM_TO_GOLGI_VESICLE_MEDIATED_TRANSPORT	0.46	2.34
GOBP_MITOCHONDRIAL_RESPIRATORY_CHAIN_COMPLEX_ASSEMBLY	0.48	2.33
GOBP_RETROGRADE_TRANSPORT_ENDOSOME_TO_GOLGI	0.54	2.3
GOBP_RIBONUCLEOPROTEIN_COMPLEX_SUBUNIT_ORGANIZATION	0.42	2.3
GOBP_TRNA_PROCESSING	0.41	2.29
GOBP_REGULATION_OF_CELL_CYCLE_PHASE_TRANSITION	0.31	2.26
GOBP_MITOTIC_CELL_CYCLE_PROCESS	0.26	2.24
GOBP_RNA_PHOSPHODIESTER_BOND_HYDROLYSIS	0.41	2.24
GOBP_MITOTIC_CELL_CYCLE	0.24	2.23
GOBP_DNA_DEPENDENT_DNA_REPLICATION	0.35	2.23
GOBP_CELL_CYCLE_PROCESS	0.23	2.21
GOBP_RNA_MODIFICATION	0.37	2.21
GOBP_REGULATION_OF_MITOTIC_CELL_CYCLE_PHASE_TRANSITION	0.33	2.21
GOBP_CHROMOSOME_ORGANIZATION	0.23	2.2
GOBP_AMIDE_BIOSYNTHETIC_PROCESS	0.24	2.18
GOBP_DNA_TEMPLATED_TRANSCRIPTION_INITIATION	0.43	2.17
GOBP_CELLULAR_RESPONSE_TO_DNA_DAMAGE_STIMULUS	0.24	2.17
GOBP_PEPTIDE_BIOSYNTHETIC_PROCESS	0.25	2.13
GOBP_MITOCHONDRIAL_TRANSLATION	0.41	2.13
GOBP_DNA_METABOLIC_PROCESS	0.22	2.11
GOBP_REGULATION_OF_DNA_REPAIR	0.44	2.1

GOBP_NUCLEAR_CHROMOSOME_SEGREGATION	0.31	2.09
GOBP_REGULATION_OF_CELLULAR_AMIDE_METABOLIC_PROCESS	0.27	2.09
GOBP_CELLULAR_MACROMOLECULE_BIOSYNTHETIC_PROCESS	0.19	2.08
GOBP_CELL_CYCLE_PHASE_TRANSITION	0.26	2.05
GOBP_CELL_CYCLE	0.19	2.05
GOBP_MITOTIC_CELL_CYCLE_PHASE_TRANSITION	0.27	1.99
GOBP_PEPTIDE_METABOLIC_PROCESS	0.21	1.97
GOBP_REGULATION_OF_CELLULAR_MACROMOLECULE_BIOSYNTHETIC_PROCESS	0.22	1.96
GOBP_MODIFICATION_DEPENDENT_MACROMOLECULE_CATABOLIC_PROCESS	0.24	1.93
GOBP_MITOCHONDRION_ORGANIZATION	0.31	2.35
GOBP_REGULATION_OF_MRNA_CATABOLIC_PROCESS	0.41	2.3
GOBP_REGULATION_OF_MRNA_METABOLIC_PROCESS	0.33	2.18
GOBP_REGULATION_OF_TRANSLATIONAL_INITIATION	0.47	2.14
GOBP_MITOCHONDRIAL_TRANSPORT	0.41	2.08
GOBP_CELL_CYCLE_CHECKPOINT_SIGNALING	0.37	2.09
GOBP_TRNA_MODIFICATION	0.42	2.02
GOBP_RECOMBINATIONAL_REPAIR	0.33	2
GOBP_INTRACELLULAR_TRANSPORT	0.19	1.95
GOBP_MITOTIC_SISTER_CHROMATID_SEGREGATION	0.34	1.95
GOBP_CELLULAR_AMIDE_METABOLIC_PROCESS	0.2	1.93
GOBP_REGULATION_OF_CYSTEINE_TYPE_ENDOPEPTIDASE_ACTIVITY	0.35	1.87
GOBP_REGULATION_OF_RESPONSE_TO_DNA_DAMAGE_STIMULUS	0.32	1.86
GOBP_POSTTRANSCRIPTIONAL_REGULATION_OF_GENE_EXPRESSION	0.23	1.85
GOBP_REGULATION_OF_CELL_CYCLE	0.19	1.83
GOBP_SISTER_CHROMATID_SEGREGATION	0.35	2.12
GOBP_RNA_DESTABILIZATION	0.44	2.09
GOBP_PROTEIN_FOLDING	0.32	2
GOBP_CYTOSOLIC_TRANSPORT	0.39	2
GOBP_RNA_SPLICING_VIA_TRANSESTERIFICATION_REACTIONS	0.3	1.99
GOBP_MATURATION_OF_SSU_RRNA	0.46	1.96
GOBP_REGULATION_OF_MITOTIC_CELL_CYCLE	0.25	1.93
GOBP_PROTEIN_TARGETING_TO_MITOCHONDRION	0.43	1.93
GOBP_RNA_SPLICING	0.27	1.92
GOBP_ESTABLISHMENT_OF_PROTEIN_LOCALIZATION_TO_ORGANELLE	0.26	1.91
GOBP_REGULATION_OF_DNA_REPLICATION	0.34	1.9
GOBP_CELLULAR_RESPIRATION	0.34	1.86
GOBP_REGULATION_OF_CELL_CYCLE_PROCESS	0.23	1.86
GOBP_PROTEIN_LOCALIZATION_TO_ORGANELLE	0.22	1.85
GOBP_NUCLEOBASE_CONTAINING_COMPOUND_TRANSPORT	0.29	1.83
GOBP_RIBOSOMAL_SMALL_SUBUNIT_BIOGENESIS	0.47	2.08
GOBP_REGULATION_OF_DOUBLE_STRAND_BREAK_REPAIR	0.45	2.02

GOBP_DNA_CONFORMATION_CHANGE	0.3	1.95
GOBP_DNA_GEOMETRIC_CHANGE	0.41	1.92
GOBP_ESTABLISHMENT_OF_RNA_LOCALIZATION	0.34	1.85
GOBP_NEGATIVE_REGULATION_OF_CELL_CYCLE_PHASE_TRANSITION	0.31	1.93
GOBP_REGULATION_OF_CHROMOSOME_SEGREGATION	0.41	1.94
GOBP_NEGATIVE_REGULATION_OF_CELLULAR_MACROMOLECULE_BIOSYNTHETIC_PROCESS	0.29	1.91
GOBP_RNA_CATABOLIC_PROCESS	0.29	1.9
GOBP_MITOTIC_CELL_CYCLE_CHECKPOINT_SIGNALING	0.36	1.87
GOBP_CELL_DIVISION	0.23	1.86
GOBP_ORGANONITROGEN_COMPOUND_BIOSYNTHETIC_PROCESS	0.16	1.67
GOBP_POSITIVE_REGULATION_OF_MITOTIC_CELL_CYCLE	0.37	1.91
GOBP_NUCLEAR_TRANSCRIBED_MRNA_CATABOLIC_PROCESS	0.38	1.81
GOBP_PROTEIN_MODIFICATION_BY_SMALL_PROTEIN_CONJUGATION_OR_REMOVAL	0.19	1.72
GOBP_ESTABLISHMENT_OF_PROTEIN_LOCALIZATION	0.15	1.55
GOBP_POSITIVE_REGULATION_OF_MRNA_METABOLIC_PROCESS	0.38	1.94
GOBP_PROTEIN_TARGETING	0.28	1.82
GOBP_MRNA_CATABOLIC_PROCESS	0.32	1.98
GOBP_RNA_3_END_PROCESSING	0.37	1.86
GOBP_ENDOSOMAL_TRANSPORT	0.31	1.76
GOBP_RIBOSOMAL_LARGE_SUBUNIT_BIOGENESIS	0.41	1.85
GOBP_RESPONSE_TO_IONIZING_RADIATION	0.4	1.86
GOBP_PROTEASOMAL_PROTEIN_CATABOLIC_PROCESS	0.22	1.67
GOBP_POSITIVE_REGULATION_OF_MITOTIC_CELL_CYCLE_PHASE_TRANSITION	0.4	1.88
GOBP_POSITIVE_REGULATION_OF_DNA_REPAIR	0.44	1.76
GOBP_SPINDLE_ORGANIZATION	0.31	1.78
GOBP_RNA_LOCALIZATION	0.3	1.77
GOBP_NEGATIVE_REGULATION_OF_MRNA_METABOLIC_PROCESS	0.43	1.94
GOBP_NUCLEIC_ACID_PHOSPHODIESTER_BOND_HYDROLYSIS	0.26	1.75
GOBP_MITOTIC_NUCLEAR_DIVISION	0.26	1.74
GOBP_PROTEIN_DNA_COMPLEX_ASSEMBLY	0.33	1.74
GOBP_NEGATIVE_REGULATION_OF_CELLULAR_AMIDE_METABOLIC_PROCESS	0.28	1.73
GOBP_PROTEIN_DNA_COMPLEX_SUBUNIT_ORGANIZATION	0.3	1.72
GOBP_ORGANELLE_FISSION	0.22	1.7
GOBP_PROTEASOME_MEDIATED_UBIQUITIN_DEPENDENT_PROTEIN_CATABOLIC_PROCESS	0.23	1.69
GOBP_AEROBIC_RESPIRATION	0.36	1.81
GOBP_PROTEIN_POLYUBIQUITINATION	0.29	1.71
GOBP_MEMBRANE_FUSION	0.36	1.77
GOBP_ORGANIC_CYCLIC_COMPOUND_CATABOLIC_PROCESS	0.22	1.65
GOBP_RNA_PHOSPHODIESTER_BOND_HYDROLYSIS_ENDONUCLEOLYTIC	0.4	1.78
GOBP_PHOSPHATIDYLINOSITOL_PHOSPHATE_BIOSYNTHETIC_PROCESS	0.41	1.67
GOBP_POSITIVE_REGULATION_OF_CELL_CYCLE_PHASE_TRANSITION	0.36	1.72

GOBP_NEGATIVE_REGULATION_OF_CELL_CYCLE_PROCESS	0.26	1.67
GOBP_NUCLEOSIDE_TRIPHOSPHATE_METABOLIC_PROCESS	0.41	1.7
GOBP_METAPHASE_ANAPHASE_TRANSITION_OF_CELL_CYCLE	0.4	1.7
GOBP_CELLULAR_AMINO_ACID_METABOLIC_PROCESS	0.25	1.69
GOBP_ORGANOPHOSPHATE_BIOSYNTHETIC_PROCESS	0.21	1.65
GOBP_PHOSPHATIDYLINOSITOL_BIOSYNTHETIC_PROCESS	0.33	1.67
GOBP_POSITIVE_REGULATION_OF_CYSTEINE_TYPE_ENDOPEPTIDASE_ACTIVITY	0.38	1.69
GOBP_ESTABLISHMENT_OF_PROTEIN_LOCALIZATION_TO_MEMBRANE	0.28	1.76
GOBP_STEROL_BIOSYNTHETIC_PROCESS	0.38	1.72
GOBP_PROTEIN_LOCALIZATION_TO_MITOCHONDRION	0.35	1.69
GOBP_CELLULAR_PROTEIN_CATABOLIC_PROCESS	0.18	1.57
GOBP_STEROID_BIOSYNTHETIC_PROCESS	0.28	1.66
GOBP_ORGANELLE_FUSION	0.37	1.71
GOBP_ORGANELLE_MEMBRANE_FUSION	0.41	1.69
GOBP_REGULATION_OF_CHROMOSOME_SEPARATION	0.4	1.7
GOBP_REGULATION_OF_CELL_CYCLE_G2_M_PHASE_TRANSITION	0.37	1.64
GOBP_DNA_RECOMBINATION	0.22	1.6
GOBP_DNA_INTEGRITY_CHECKPOINT_SIGNALING	0.36	1.8
GOBP_MICROTUBULE_ORGANIZING_CENTER_ORGANIZATION	0.4	1.74
GOBP_TRANSCRIPTION_BY_RNA_POLYMERASE_I	0.39	1.62
GOBP_NUCLEAR_EXPORT	0.29	1.6
GOBP_POSITIVE_REGULATION_OF_CELL_CYCLE_PROCESS	0.28	1.67
GOBP_CHROMOSOME_SEPARATION	0.34	1.67
GOBP_INTRACELLULAR_PROTEIN_TRANSPORT	0.17	1.53
GOBP_POSITIVE_REGULATION_OF_VIRAL_PROCESS	0.39	1.65
GOBP_REGULATION_OF_DNA_DEPENDENT_DNA_REPLICATION	0.39	1.64
GOBP_PROTEIN_MODIFICATION_BY_SMALL_PROTEIN_CONJUGATION	0.18	1.5
GOBP_CELLULAR_NITROGEN_COMPOUND_CATABOLIC_PROCESS	0.21	1.56
GOBP_RESPONSE_TO_ENDOPLASMIC_RETICULUM_STRESS	0.24	1.56
GOBP_REGULATION_OF_RESPONSE_TO_ENDOPLASMIC_RETICULUM_STRESS	0.39	1.6

Table S2. Biological processes in the Gene Ontology (GO) analysis of downregulated gene sets in nobiletin-treated HepG2 cells.

Gene Sets Follow link to MsigDB	Enrichment Score (ES)	Nominal ES (NES)
GOBP_BIOLOGICAL_ADHESION	-0.38	-3.86
GOBP_ANIMAL_ORGAN_MORPHOGENESIS	-0.38	-3.38
GOBP_REGULATION_OF_CELL_ADHESION	-0.38	-3.35
GOBP_CELL_MIGRATION	-0.32	-3.23
GOBP_POSITIVE_REGULATION_OF_CELL_CELL_ADHESION	-0.5	-3.22
GOBP_REGULATION_OF_CELL_CELL_ADHESION	-0.42	-3.21
GOBP_GLIOGENESIS	-0.53	-3.17
GOBP_GLIAL_CELL_DIFFERENTIATION	-0.56	-3.16
GOBP_POSITIVE_REGULATION_OF_CELL_ADHESION	-0.44	-3.16
GOBP_CELL_CELL_ADHESION	-0.36	-3.15
GOBP_LOCOMOTION	-0.31	-3.15
GOBP_PEPTIDYL_TYROSINE_MODIFICATION	-0.49	-3.14
GOBP_TAXIS	-0.37	-3.1
GOBP_NEURON_PROJECTION_GUIDANCE	-0.53	-3.09
GOBP_CELL_MORPHOGENESIS_INVOLVED_IN_DIFFERENTIATION	-0.38	-3.09
GOBP_NEGATIVE_REGULATION_OF_MULTICELLULAR_ORGANISMAL_PROCESS	-0.34	-3.08
GOBP_EXTERNAL_ENCAPSULATING_STRUCTURE_ORGANIZATION	-0.47	-3.08
GOBP_POSITIVE_REGULATION_OF_LEUKOCYTE_CELL_CELL_ADHESION	-0.51	-3.05
GOBP_NEUROGENESIS	-0.3	-3.04
GOBP_REGULATION_OF_CELL_ACTIVATION	-0.38	-3.02
GOBP_POSITIVE_REGULATION_OF_CELL_ACTIVATION	-0.44	-2.99
GOBP_SODIUM_ION_TRANSPORT	-0.53	-2.98
GOBP_REGULATION_OF_PEPTIDYL_TYROSINE_PHOSPHORYLATION	-0.52	-2.98
GOBP_ANATOMICAL_STRUCTURE_FORMATION_INVOLVED_IN_MORPHOGENESIS	-0.31	-2.97
GOBP_BLOOD_VESSEL_MORPHOGENESIS	-0.35	-2.96
GOBP_AXON_DEVELOPMENT	-0.41	-2.96
GOBP_POSITIVE_REGULATION_OF_CELL_DIFFERENTIATION	-0.33	-2.92
GOBP_LEUKOCYTE_CELL_CELL_ADHESION	-0.41	-2.92
GOBP_DEFENSE_RESPONSE_TO_BACTERIUM	-0.48	-2.91
GOBP_DEFENSE_RESPONSE	-0.28	-2.91
GOBP_REGULATION_OF_CELL_DIFFERENTIATION	-0.29	-2.9
GOBP_REGULATION_OF_CELL_POPULATION_PROLIFERATION	-0.28	-2.89
GOBP_MORPHOGENESIS_OF_AN_EPITHELIUM	-0.38	-2.88
GOBP_REGULATION_OF_CELLULAR_COMPONENT_MOVEMENT	-0.31	-2.87
GOBP_CELL_MORPHOGENESIS	-0.32	-2.87
GOBP_HUMORAL_IMMUNE_RESPONSE	-0.46	-2.87
GOBP_CARBOXYLIC_ACID_TRANSPORT	-0.45	-2.87

GOBP_INFLAMMATORY_RESPONSE	-0.33	-2.85
GOBP_NEURON_DIFFERENTIATION	-0.29	-2.83
GOBP_ORGANIC_ACID_TRANSPORT	-0.42	-2.83
GOBP_IMMUNE_RESPONSE	-0.28	-2.81
GOBP_RESPONSE_TO_BACTERIUM	-0.35	-2.81
GOBP_NEGATIVE_REGULATION_OF_CELL_POPULATION_PROLIFERATION	-0.33	-2.8
GOBP_RESPONSE_TO_LIPID	-0.31	-2.79
GOBP_METAL_ION_TRANSPORT	-0.34	-2.79
GOBP_ENZYME_LINKED_RECEPTOR_PROTEIN_SIGNALING_PATHWAY	-0.31	-2.78
GOBP_REGULATION_OF_MULTICELLULAR_ORGANISMAL_DEVELOPMENT	-0.28	-2.78
GOBP_RESPONSE_TO_RETINOIC_ACID	-0.59	-2.78
GOBP_REGULATION_OF_LYMPHOCYTE_ACTIVATION	-0.37	-2.77
GOBP_TUBE_MORPHOGENESIS	-0.31	-2.77
GOBP_ION_TRANSPORT	-0.29	-2.77
GOBP_CELL_MORPHOGENESIS_INVOLVED_IN_NEURON_DIFFERENTIATION	-0.38	-2.77
GOBP_POSITIVE_REGULATION_OF_IMMUNE_SYSTEM_PROCESS	-0.31	-2.76
GOBP_TISSUE_DEVELOPMENT	-0.26	-2.74
GOBP_TUBE_DEVELOPMENT	-0.29	-2.74
GOBP_NEGATIVE_REGULATION_OF_RESPONSE_TO_WOUNDING	-0.63	-2.74
GOBP_CELL_JUNCTION_ORGANIZATION	-0.34	-2.73
GOBP_G_PROTEIN_COUPLED_RECEPTOR_SIGNALING_PATHWAY	-0.35	-2.72
GOBP_REGULATION_OF_T_CELL_ACTIVATION	-0.39	-2.72
GOBP_CIRCULATORY_SYSTEM_DEVELOPMENT	-0.28	-2.71
GOBP_REGULATION_OF_ANATOMICAL_STRUCTURE_MORPHOGENESIS	-0.3	-2.71
GOBP_CIRCULATORY_SYSTEM_PROCESS	-0.36	-2.71
GOBP_POSITIVE_REGULATION_OF_DEVELOPMENTAL_PROCESS	-0.28	-2.7
GOBP_NEURON_DEVELOPMENT	-0.29	-2.69
GOBP_CELL_CELL_SIGNALING	-0.28	-2.69
GOBP_POSITIVE_REGULATION_OF_MULTICELLULAR_ORGANISMAL_PROCESS	-0.28	-2.69
GOBP_NEGATIVE_REGULATION_OF_WOUND_HEALING	-0.63	-2.69
GOBP_ORGANIC_HYDROXY_COMPOUND_TRANSPORT	-0.42	-2.69
GOBP_REGULATION_OF_TRANSMEMBRANE_TRANSPORT	-0.36	-2.68
GOBP_ERK1_AND_ERK2_CASCADE	-0.41	-2.67
GOBP_ADAPTIVE_IMMUNE_RESPONSE	-0.37	-2.67
GOBP_ANION_TRANSPORT	-0.35	-2.67
GOBP_VASCULATURE_DEVELOPMENT	-0.31	-2.66
GOBP_OSTEOCLAST_DIFFERENTIATION	-0.63	-2.66
GOBP_NEGATIVE_REGULATION_OF_COAGULATION	-0.63	-2.65
GOBP_REGULATION_OF_IMMUNE_SYSTEM_PROCESS	-0.26	-2.65
GOBP_POSITIVE_REGULATION_OF_PEPTIDYL_TYROSINE_PHOSPHORYLATION	-0.53	-2.64
GOBP_CELL_SUBSTRATE_ADHESION	-0.36	-2.64

**GOBP_POSITIVE_REGULATION_OF_ION_TRANSPORT**	**-0.44**	**-2.62**
**GOBP_POSITIVE_REGULATION_OF_CELL_JUNCTION_ASSEMBLY**	**-0.62**	**-2.62**
**GOBP_EPITHELIAL_CELL_PROLIFERATION**	**-0.36**	**-2.61**
**GOBP_POSITIVE_REGULATION_OF_TRANSPORT**	**-0.3**	**-2.61**
**GOBP_SODIUM_ION_TRANSMEMBRANE_TRANSPORT**	**-0.54**	**-2.6**
**GOBP_REGULATION_OF_ION_TRANSPORT**	**-0.34**	**-2.6**
**GOBP_NEGATIVE_REGULATION_OF_LOCOMOTION**	**-0.39**	**-2.59**
**GOBP_IMMUNE_EFFECTOR_PROCESS**	**-0.32**	**-2.58**
**GOBP_ORGANIC_ANION_TRANSPORT**	**-0.37**	**-2.58**
**GOBP_TISSUE_MORPHOGENESIS**	**-0.32**	**-2.56**
**GOBP_LIPID_LOCALIZATION**	**-0.34**	**-2.56**
**GOBP_CELL_ACTIVATION**	**-0.27**	**-2.55**
**GOBP_POSITIVE_REGULATION_OF_SECRETION**	**-0.39**	**-2.55**
**GOBP_REGULATION_OF_RESPONSE_TO_EXTERNAL_STIMULUS**	**-0.28**	**-2.55**
**GOBP_HUMORAL_IMMUNE_RESPONSE_MEDIATED_BY_CIRCULATING_IMMUNOGLOBULIN**	**-0.59**	**-2.54**
**GOBP_NEGATIVE_REGULATION_OF_RESPONSE_TO_EXTERNAL_STIMULUS**	**-0.36**	**-2.54**
**GOBP_REGULATION_OF_IMMUNE_EFFECTOR_PROCESS**	**-0.38**	**-2.53**
**GOBP_DEFENSE_RESPONSE_TO_GRAM_POSITIVE_BACTERIUM**	**-0.61**	**-2.53**
**GOBP_NEGATIVE_REGULATION_OF_CYTOKINE_PRODUCTION**	**-0.43**	**-2.52**
**GOBP_CELL_PART_MORPHOGENESIS**	**-0.32**	**-2.52**
**GOBP_CELLULAR_COMPONENT_MORPHOGENESIS**	**-0.32**	**-2.52**
**GOBP_REGULATION_OF_SECRETION**	**-0.32**	**-2.52**
**GOBP_REGULATION_OF_RESPONSE_TO_WOUNDING**	**-0.47**	**-2.52**
**GOBP_POSITIVE_REGULATION_OF_PHOSPHORUS_METABOLIC_PROCESS**	**-0.28**	**-2.52**
**GOBP_COLLAGEN_METABOLIC_PROCESS**	**-0.55**	**-2.51**
**GOBP_REGULATION_OF_SIGNALING_RECEPTOR_ACTIVITY**	**-0.54**	**-2.51**
**GOBP_INTERLEUKIN_6_PRODUCTION**	**-0.52**	**-2.51**
**GOBP_POSITIVE_REGULATION_OF_CELL_POPULATION_PROLIFERATION**	**-0.28**	**-2.51**
**GOBP_GLIAL_CELL_DEVELOPMENT**	**-0.57**	**-2.51**
**GOBP_REGULATION_OF_IMMUNE_RESPONSE**	**-0.28**	**-2.5**
**GOBP_CATION_TRANSPORT**	**-0.28**	**-2.49**
**GOBP_SYNAPSE_ORGANIZATION**	**-0.37**	**-2.49**
**GOBP_NERVOUS_SYSTEM_PROCESS**	**-0.29**	**-2.48**
**GOBP_NEGATIVE_REGULATION_OF_CELL_DIFFERENTIATION**	**-0.31**	**-2.48**
**GOBP_NEGATIVE_REGULATION_OF_CELL_ADHESION**	**-0.39**	**-2.48**
**GOBP_EPITHELIUM_DEVELOPMENT**	**-0.26**	**-2.48**
**GOBP_REGULATION_OF_CELL_JUNCTION_ASSEMBLY**	**-0.47**	**-2.48**
**GOBP_HORMONE_METABOLIC_PROCESS**	**-0.39**	**-2.47**
**GOBP_RESPONSE_TO_OXYGEN_CONTAINING_COMPOUND**	**-0.24**	**-2.47**
**GOBP_REGULATION_OF_LEUKOCYTE_DIFFERENTIATION**	**-0.38**	**-2.46**
**GOBP_ODONTOGENESIS**	**-0.56**	**-2.45**

GOBP_REGULATION_OF_ION_TRANSMEMBRANE_TRANSPORT	-0.35	-2.44
GOBP_ANATOMICAL_STRUCTURE_HOMEOSTASIS	-0.38	-2.43
GOBP_T_CELL_ACTIVATION	-0.31	-2.43
GOBP_NEGATIVE_REGULATION_OF_CELL_DEVELOPMENT	-0.49	-2.42
GOBP_REGULATION_OF_BLOOD_PRESSURE	-0.48	-2.42
GOBP_AMEBOIDAL_TYPE_CELL_MIGRATION	-0.33	-2.42
GOBP_POSITIVE_REGULATION_OF_MAPK_CASCADE	-0.32	-2.41
GOBP_REGULATION_OF_HORMONE_LEVELS	-0.31	-2.4
GOBP_LYMPHOCYTE_ACTIVATION	-0.28	-2.4
GOBP_CELL_JUNCTION_ASSEMBLY	-0.35	-2.4
GOBP_POSITIVE_REGULATION_OF_IMMUNE_RESPONSE	-0.3	-2.4
GOBP_EMBRYONIC_MORPHOGENESIS	-0.29	-2.37
GOBP_CELL_CELL_ADHESION_VIA_PLASMA_MEMBRANE_ADHESION_MOLECULES	-0.43	-2.37
GOBP_LEUKOCYTE_DIFFERENTIATION	-0.3	-2.37
GOBP_DETECTION_OF_STIMULUS	-0.38	-2.36
GOBP_CELL_MATRIX_ADHESION	-0.38	-2.36
GOBP_POSITIVE_REGULATION_OF_ERK1_AND_ERK2_CASCADE	-0.43	-2.36
GOBP_LYMPHOCYTE_MEDIATED_IMMUNITY	-0.37	-2.36
GOBP_REGULATION_OF_NERVOUS_SYSTEM_DEVELOPMENT	-0.35	-2.36
GOBP_CELLULAR_RESPONSE_TO_LIPID	-0.32	-2.35
GOBP_LEUKOCYTE_PROLIFERATION	-0.36	-2.35
GOBP_ACTIVATION_OF_IMMUNE_RESPONSE	-0.36	-2.35
GOBP_MORPHOGENESIS_OF_A_BRANCHING_STRUCTURE	-0.44	-2.34
GOBP_CELLULAR_RESPONSE_TO_OXYGEN_CONTAINING_COMPOUND	-0.25	-2.34
GOBP_SKELETAL_SYSTEM_DEVELOPMENT	-0.31	-2.34
GOBP_B_CELL_MEDIATED_IMMUNITY	-0.42	-2.34
GOBP_NEGATIVE_REGULATION_OF_DEVELOPMENTAL_PROCESS	-0.26	-2.34
GOBP_BIOMINERALIZATION	-0.48	-2.33
GOBP_RESPONSE_TO_MOLECULE_OF_BACTERIAL_ORIGIN	-0.36	-2.33
GOBP_SYNAPSE_ASSEMBLY	-0.5	-2.33
GOBP_REGULATION_OF_PROTEIN_PHOSPHORYLATION	-0.25	-2.32
GOBP_HEART_MORPHOGENESIS	-0.38	-2.32
GOBP_OSSIFICATION	-0.33	-2.32
GOBP_POSITIVE_REGULATION_OF_PROTEIN_PHOSPHORYLATION	-0.27	-2.31
GOBP_ARTERY_DEVELOPMENT	-0.47	-2.31
GOBP_POSITIVE_REGULATION_OF_CELL_DEVELOPMENT	-0.38	-2.3
GOBP_NEGATIVE_REGULATION_OF_CELL_CELL_ADHESION	-0.4	-2.3
GOBP_TRANSMEMBRANE_RECEPTOR_PROTEIN_TYROSINE _KINASE_SIGNALING_PATHWAY	-0.3	-2.3
GOBP_HORMONE_TRANSPORT	-0.36	-2.3
GOBP_REGULATION_OF_SYNAPSE_STRUCTURE_OR_ACTIVITY	-0.43	-2.3
GOBP_REGULATION_OF_CELL_SUBSTRATE_ADHESION	-0.39	-2.29

GOBP_POSITIVE_REGULATION_OF_IMMUNE_EFFECTOR_PROCESS	-0.38	-2.29
GOBP_POSITIVE_REGULATION_OF_LOCOMOTION	-0.28	-2.28
GOBP_HEART_DEVELOPMENT	-0.28	-2.28
GOBP_REGULATION_OF_PHOSPHORUS_METABOLIC_PROCESS	-0.23	-2.28
GOBP_MACROPHAGE_ACTIVATION	-0.53	-2.28
GOBP_VASCULAR_PROCESS_IN_CIRCULATORY_SYSTEM	-0.36	-2.27
GOBP_LEUKOCYTE_MEDIATED_IMMUNITY	-0.32	-2.27
GOBP_REGULATION_OF_CELL_PROJECTION_ORGANIZATION	-0.29	-2.26
GOBP_STEROL_HOMEOSTASIS	-0.5	-2.26
GOBP_REGULATION_OF_WOUND_HEALING	-0.44	-2.26
GOBP_EXPORT_FROM_CELL	-0.26	-2.25
GOBP_CELL_SURFACE_RECEPTOR_SIGNALING_PATHWAY _INVOLVED_IN_CELL_CELL_SIGNALING	-0.3	-2.25
GOBP_RESPONSE_TO_ALCOHOL	-0.38	-2.24
GOBP_REGULATION_OF_CELL_DEVELOPMENT	-0.32	-2.24
GOBP_REGULATION_OF_ANIMAL_ORGAN_MORPHOGENESIS	-0.52	-2.24
GOBP_ENDODERM_DEVELOPMENT	-0.54	-2.24
GOBP_REGULATION_OF_LEUKOCYTE_PROLIFERATION	-0.37	-2.24
GOBP_CELL_PROJECTION_ORGANIZATION	-0.24	-2.24
GOBP_INORGANIC_ION_TRANSMEMBRANE_TRANSPORT	-0.27	-2.23
GOBP_ION_TRANSMEMBRANE_TRANSPORT	-0.26	-2.23
GOBP_APPENDAGE_DEVELOPMENT	-0.42	-2.23
GOBP_REGULATION_OF_HEMOPOIESIS	-0.31	-2.23
GOBP_POSITIVE_REGULATION_OF_TRANSMEMBRANE_TRANSPORT	-0.43	-2.23
GOBP_SECRETION	-0.25	-2.21
GOBP_DEFENSE_RESPONSE_TO_OTHER_ORGANISM	-0.24	-2.21
GOBP_BEHAVIOR	-0.3	-2.2
GOBP_NEGATIVE_REGULATION_OF_CELL_ACTIVATION	-0.39	-2.2
GOBP_BIOLOGICAL_PROCESS_INVOLVED_IN_INTERSPECIES _INTERACTION_BETWEEN_ORGANISMS	-0.22	-2.2
GOBP_REGULATION_OF_MYELOID_LEUKOCYTE_DIFFERENTIATION	-0.45	-2.2
GOBP_ENDOTHELIAL_CELL_MIGRATION	-0.38	-2.19
GOBP_RESPONSE_TO_MECHANICAL_STIMULUS	-0.38	-2.19
GOBP_SIGNAL_RELEASE	-0.31	-2.19
GOBP_REGULATION_OF_PHOSPHATIDYLINOSITOL_3_KINASE_SIGNALING	-0.52	-2.18
GOBP_STRIATED_MUSCLE_CONTRACTION	-0.49	-2.18
GOBP_INORGANIC_ANION_TRANSPORT	-0.41	-2.18
GOBP_RESPONSE_TO_WOUNDING	-0.26	-2.18
GOBP_POSITIVE_REGULATION_OF_CELLULAR_COMPONENT_BIOGENESIS	-0.31	-2.17
GOBP_REGULATION_OF_LEUKOCYTE_MEDIATED_IMMUNITY	-0.37	-2.17
GOBP_REGULATION_OF_T_CELL_DIFFERENTIATION	-0.42	-2.17
GOBP_MAPK_CASCADE	-0.26	-2.17
GOBP_APPENDAGE_MORPHOGENESIS	-0.41	-2.17

GOBP_EPIDERMIS_DEVELOPMENT	-0.31	-2.17
GOBP_EPITHELIAL_CELL_DIFFERENTIATION	-0.25	-2.16
GOBP_MULTICELLULAR_ORGANISMAL_HOMEOSTASIS	-0.3	-2.16
GOBP_REGULATION_OF_OSSIFICATION	-0.44	-2.14
GOBP_REGULATION_OF_CYTOSOLIC_CALCIUM_ION_CONCENTRATION	-0.34	-2.13
GOBP_EMBRYONIC_ORGAN_DEVELOPMENT	-0.28	-2.11
GOBP_RESPONSE_TO_OXYGEN_LEVELS	-0.32	-2.11
GOBP_KIDNEY_EPITHELIUM_DEVELOPMENT	-0.47	-2.11
GOBP_TRANSMEMBRANE_TRANSPORT	-0.22	-2.11
GOBP_REGULATION_OF_ANATOMICAL_STRUCTURE_SIZE	-0.28	-2.1
GOBP_REGULATION_OF_METAL_ION_TRANSPORT	-0.33	-2.1
GOBP_ENTRY_INTO_HOST	-0.43	-2.1
GOBP_NEGATIVE_REGULATION_OF_MOLECULAR_FUNCTION	-0.23	-2.1
GOBP_POSITIVE_REGULATION_OF_KINASE_ACTIVITY	-0.28	-2.1
GOBP_IMMUNE_RESPONSE_REGULATING_CELL_ SURFACE_RECEPTOR_SIGNALING_PATHWAY	-0.37	-2.1
GOBP_EYE_MORPHOGENESIS	-0.44	-2.1
GOBP_REGULATION_OF_NEURON_PROJECTION_DEVELOPMENT	-0.31	-2.09
GOBP_INTERFERON_GAMMA_PRODUCTION	-0.5	-2.09
GOBP_REGULATION_OF_TRANSPORTER_ACTIVITY	-0.36	-2.09
GOBP_COGNITION	-0.35	-2.08
GOBP_HOMEOSTATIC_PROCESS	-0.2	-2.08
GOBP_GLAND_MORPHOGENESIS	-0.41	-2.08
GOBP_POSITIVE_REGULATION_OF_SIGNALING	-0.21	-2.08
GOBP_CYTOKINE_PRODUCTION	-0.26	-2.08
GOBP_EPITHELIAL_TUBE_MORPHOGENESIS	-0.31	-2.06
GOBP_ENDOTHELIAL_CELL_PROLIFERATION	-0.39	-2.05
GOBP_REGULATION_OF_HYDROLASE_ACTIVITY	-0.22	-2.03
GOBP_SECOND_MESSENGER_MEDIATED_SIGNALING	-0.35	-2
GOBP_RESPONSE_TO_ENDOGENOUS_STIMULUS	-0.19	-1.98
GOBP_RESPONSE_TO_INTERFERON_GAMMA	-0.41	-1.98
GOBP_NEGATIVE_REGULATION_OF_RESPONSE_TO_STIMULUS	-0.2	-1.96
GOBP_POSITIVE_REGULATION_OF_CELLULAR_COMPONENT_ORGANIZATION	-0.22	-1.93
GOBP_NEGATIVE_REGULATION_OF_SECRETION	-0.4	-1.9
GOBP_CELLULAR_RESPONSE_TO_ENDOGENOUS_STIMULUS	-0.19	-1.86
GOBP_REGULATION_OF_PROTEIN_MODIFICATION_PROCESS	-0.17	-1.76
GOBP_POTASSIUM_ION_TRANSPORT	-0.59	-2.84
GOBP_NEGATIVE_REGULATION_OF_NERVOUS_SYSTEM_DEVELOPMENT	-0.51	-2.46
GOBP_CARDIOCYTE_DIFFERENTIATION	-0.49	-2.42
GOBP_SENSORY_PERCEPTION	-0.32	-2.37
GOBP_REGULATION_OF_MAP_KINASE_ACTIVITY	-0.46	-2.35
GOBP_POSITIVE_REGULATION_OF_HEMOPOIESIS	-0.46	-2.35

GOBP_REGULATION_OF_FAT_CELL_DIFFERENTIATION	-0.48	-2.32
GOBP_COMPLEMENT_ACTIVATION	-0.53	-2.28
GOBP_RESPONSE_TO_ETHANOL	-0.53	-2.28
GOBP_SENSORY_PERCEPTION_OF_CHEMICAL_STIMULUS	-0.55	-2.28
GOBP_POSITIVE_REGULATION_OF_NERVOUS_SYSTEM_DEVELOPMENT	-0.39	-2.26
GOBP_ACTIN_FILAMENT_BASED_PROCESS	-0.27	-2.25
GOBP_FORMATION_OF_PRIMARY_GERM_LAYER	-0.44	-2.24
GOBP_EPITHELIAL_CELL_DEVELOPMENT	-0.37	-2.24
GOBP_ARTERY_MORPHOGENESIS	-0.5	-2.24
GOBP_POSITIVE_REGULATION_OF_LIPID_METABOLIC_PROCESS	-0.47	-2.23
GOBP_IMPORT_ACROSS_PLASMA_MEMBRANE	-0.41	-2.2
GOBP_POSITIVE_REGULATION_OF_HORMONE_SECRETION	-0.48	-2.2
GOBP_CELL_CHEMOTAXIS	-0.33	-2.19
GOBP_MYELOID_LEUKOCYTE_DIFFERENTIATION	-0.36	-2.19
GOBP_ENDOCYTOSIS	-0.29	-2.18
GOBP_CELLULAR_RESPONSE_TO_INTERFERON_GAMMA	-0.48	-2.17
GOBP_VASCULOGENESIS	-0.46	-2.17
GOBP_RESPONSE_TO_CORTICOSTEROID	-0.43	-2.16
GOBP_IMPORT_INTO_CELL	-0.39	-2.16
GOBP_ADAPTIVE_IMMUNE_RESPONSE_BASED_ON_SOMATIC _RECOMBINATION_OF_IMMUNE_RECEPTORS_BUILT_FROM_ IMMUNOGLOBULIN_SUPERFAMILY_DOMAINS	-0.33	-2.16
GOBP_T_CELL_PROLIFERATION	-0.37	-2.16
GOBP_MONOCARBOXYLIC_ACID_TRANSPORT	-0.49	-2.16
GOBP_RESPONSE_TO_INTERLEUKIN_1	-0.48	-2.16
GOBP_CELL_FATE_COMMITMENT	-0.38	-2.15
GOBP_PATTERN_SPECIFICATION_PROCESS	-0.3	-2.14
GOBP_AMINO_ACID_TRANSPORT	-0.45	-2.13
GOBP_REGULATION_OF_CHEMOTAXIS	-0.35	-2.13
GOBP_TISSUE_REMODELING	-0.42	-2.11
GOBP_DICARBOXYLIC_ACID_TRANSPORT	-0.48	-2.11
GOBP_POSITIVE_REGULATION_OF_EPITHELIAL_CELL_PROLIFERATION	-0.38	-2.11
GOBP_SENSORY_ORGAN_DEVELOPMENT	-0.27	-2.1
GOBP_NEGATIVE_REGULATION_OF_PEPTIDASE_ACTIVITY	-0.35	-2.1
GOBP_CELLULAR_RESPONSE_TO_INTERLEUKIN_1	-0.46	-2.1
GOBP_SENSORY_ORGAN_MORPHOGENESIS	-0.36	-2.09
GOBP_REGULATION_OF_MEMBRANE_POTENTIAL	-0.35	-2.09
GOBP_REGULATION_OF_TRANSMEMBRANE_RECEPTOR _PROTEIN_SERINE_THREONINE_KINASE_SIGNALING_PATHWAY	-0.34	-2.08
GOBP_INNATE_IMMUNE_RESPONSE	-0.25	-2.08
GOBP_PHOSPHATIDYLINOSITOL_3_KINASE_SIGNALING	-0.43	-2.06
GOBP_REGULATION_OF_HORMONE_SECRETION	-0.36	-2.06
GOBP_ANION_TRANSMEMBRANE_TRANSPORT	-0.35	-2.06

GOBP_HEART_PROCESS	-0.41	-2.05
GOBP_RESPONSE_TO_XENOBIOTIC_STIMULUS	-0.28	-2.05
GOBP_CANONICAL_WNT_SIGNALING_PATHWAY	-0.34	-2.04
GOBP_CELLULAR_RESPONSE_TO_BIOTIC_STIMULUS	-0.34	-2.03
GOBP_POSITIVE_REGULATION_OF_INTERLEUKIN_6_PRODUCTION	-0.49	-2.03
GOBP_REGULATION_OF_CELL_MORPHOGENESIS	-0.33	-2.02
GOBP_NEGATIVE_REGULATION_OF_CATALYTIC_ACTIVITY	-0.24	-2.01
GOBP_MONONUCLEAR_CELL_DIFFERENTIATION	-0.27	-2.01
GOBP_CATION_TRANSMEMBRANE_TRANSPORT	-0.25	-2
GOBP_MUSCLE_CELL_PROLIFERATION	-0.34	-1.98
GOBP_REGULATION_OF_EPITHELIAL_CELL_MIGRATION	-0.32	-1.98
GOBP_EMBRYONIC_ORGAN_MORPHOGENESIS	-0.32	-1.97
GOBP_INOSITOL_LIPID_MEDIATED_SIGNALING	-0.37	-1.96
GOBP_ACTIN_FILAMENT_BUNDLE_ORGANIZATION	-0.43	-1.95
GOBP_CALCIUM_MEDIATED_SIGNALING	-0.42	-1.94
GOBP_SUBSTRATE_ADHESION_DEPENDENT_CELL_SPREADING	-0.41	-1.94
GOBP_CENTRAL_NERVOUS_SYSTEM_DEVELOPMENT	-0.22	-1.94
GOBP_CHEMICAL_HOMEOSTASIS	-0.21	-1.93
GOBP_REGULATION_OF_TRANSPORT	-0.19	-1.93
GOBP_REGULATION_OF_SYSTEM_PROCESS	-0.27	-1.92
GOBP_CELL_CELL_SIGNALING_BY_WNT	-0.27	-1.92
GOBP_CELLULAR_HOMEOSTASIS	-0.23	-1.92
GOBP_TISSUE_MIGRATION	-0.29	-1.91
GOBP_NEGATIVE_REGULATION_OF_HYDROLASE_ACTIVITY	-0.27	-1.9
GOBP_PHOSPHORYLATION	-0.18	-1.9
GOBP_POSITIVE_REGULATION_OF_INTRACELLULAR_SIGNAL_TRANSDUCTION	-0.2	-1.88
GOBP_UROGENITAL_SYSTEM_DEVELOPMENT	-0.29	-1.86
GOBP_RESPONSE_TO_HORMONE	-0.21	-1.83
GOBP_SENSORY_SYSTEM_DEVELOPMENT	-0.27	-1.81
GOBP_REGULATION_OF_LYMPHOCYTE_DIFFERENTIATION	-0.41	-2.24
GOBP_POSITIVE_REGULATION_OF_EXOCYTOSIS	-0.53	-2.2
GOBP_CELLULAR_HORMONE_METABOLIC_PROCESS	-0.43	-2.2
GOBP_ACTION_POTENTIAL	-0.53	-2.2
GOBP_REGULATION_OF_COAGULATION	-0.45	-2.16
GOBP_FAT_CELL_DIFFERENTIATION	-0.35	-2.14
GOBP_POSITIVE_REGULATION_OF_MAP_KINASE_ACTIVITY	-0.48	-2.14
GOBP_MULTI_MULTICELLULAR_ORGANISM_PROCESS	-0.36	-2.13
GOBP_REGULATION_OF_LIPID_LOCALIZATION	-0.38	-2.12
GOBP_REGULATION_OF_T_CELL_PROLIFERATION	-0.39	-2.11
GOBP_SPECIFICATION_OF_SYMMETRY	-0.45	-2.09
GOBP_REGULATION_OF_NEUROGENESIS	-0.32	-2.09

GOBP_REGULATION_OF_HEART_CONTRACTION	-0.45	-2.09
GOBP_HOMOPHILIC_CELL_ADHESION_VIA_PLASMA_MEMBRANE_ADHESION_MOLECULES	-0.48	-2.08
GOBP_CELLULAR_RESPONSE_TO_MOLECULE_OF_BACTERIAL_ORIGIN	-0.38	-2.07
GOBP_REGULATION_OF_BLOOD_CIRCULATION	-0.38	-2.06
GOBP_CENTRAL_NERVOUS_SYSTEM_NEURON_DIFFERENTIATION	-0.45	-2.06
GOBP_IMMUNE_RESPONSE_REGULATING_SIGNALING_PATHWAY	-0.29	-2.04
GOBP_CARDIAC_SEPTUM_DEVELOPMENT	-0.43	-2.04
GOBP_PHAGOCYTOSIS	-0.31	-2.03
GOBP_REGULATION_OF_GTPASE_ACTIVITY	-0.29	-2.03
GOBP_NEGATIVE_REGULATION_OF_EPITHELIAL_CELL_MIGRATION	-0.47	-2.02
GOBP_ORGANIC_ACID_TRANSMEMBRANE_TRANSPORT	-0.38	-2
GOBP_REGULATION_OF_EXOCYTOSIS	-0.37	-1.99
GOBP_NEUTROPHIL_MIGRATION	-0.4	-1.98
GOBP_LEUKOCYTE_MIGRATION	-0.28	-1.97
GOBP_NEGATIVE_REGULATION_OF_PROTEOLYSIS	-0.3	-1.97
GOBP_TRANSMEMBRANE_RECEPTOR_PROTEIN_SERINE_THREONINE_KINASE_SIGNALING_PATHWAY	-0.3	-1.97
GOBP_STEROL_TRANSPORT	-0.4	-1.97
GOBP_EMBRYONIC_APPENDAGE_MORPHOGENESIS	-0.41	-1.96
GOBP_CARDIAC_MUSCLE_CELL_DIFFERENTIATION	-0.47	-1.95
GOBP_SMOOTH_MUSCLE_CELL_PROLIFERATION	-0.38	-1.95
GOBP_MYELOID_LEUKOCYTE_ACTIVATION	-0.32	-1.88
GOBP_RESPONSE_TO_PEPTIDE	-0.25	-1.85
GOBP_POSITIVE_REGULATION_OF_PROTEIN_MODIFICATION_PROCESS	-0.2	-1.8
GOBP_RESPONSE_TO_BMP	-0.39	-2.08
GOBP_CELL_CELL_JUNCTION_ORGANIZATION	-0.41	-2.07
GOBP_REGULATION_OF_BIOMINERALIZATION	-0.49	-2.04
GOBP_REGULATION_OF_ENDOTHELIAL_CELL_MIGRATION	-0.38	-2.03
GOBP_REGULATION_OF_TUBE_SIZE	-0.4	-2.03
GOBP_DIGESTION	-0.4	-2
GOBP_REGENERATION	-0.35	-1.99
GOBP_REGULATION_OF_VASCULATURE_DEVELOPMENT	-0.3	-1.98
GOBP_POSITIVE_REGULATION_OF_T_CELL_PROLIFERATION	-0.43	-1.97
GOBP_POSITIVE_REGULATION_OF_NEUROGENESIS	-0.36	-1.95
GOBP_SPROUTING_ANGIOGENESIS	-0.42	-1.94
GOBP_CALCIUM_ION_TRANSPORT	-0.3	-1.92
GOBP_T_CELL_DIFFERENTIATION	-0.29	-1.91
GOBP_MESENCHYMAL_CELL_DEVELOPMENT	-0.43	-1.91
GOBP_POSITIVE_REGULATION_OF_ION_TRANSMEMBRANE_TRANSPORT	-0.39	-1.91
GOBP_POSITIVE_REGULATION_OF_SMALL_MOLECULE_METABOLIC_PROCESS	-0.41	-1.9
GOBP_CELL_FATE_SPECIFICATION	-0.43	-1.89
GOBP_RESPONSE_TO_CALCIUM_ION	-0.37	-1.89

GOBP_CELLULAR_RESPONSE_TO_XENOBIOTIC_STIMULUS	-0.36	-1.89
GOBP_REGULATION_OF_CATION_CHANNEL_ACTIVITY	-0.43	-1.86
GOBP_REGULATION_OF_CELLULAR_COMPONENT_BIOGENESIS	-0.21	-1.84
GOBP_REGULATION_OF_JNK_CASCADE	-0.36	-1.81
GOBP_MONOCARBOXYLIC_ACID_METABOLIC_PROCESS	-0.22	-1.81
GOBP_STEROID_METABOLIC_PROCESS	-0.25	-1.8
GOBP_STRIATED_MUSCLE_CELL_DIFFERENTIATION	-0.32	-1.8
GOBP_BLOOD_VESSEL_ENDOTHELIAL_CELL_MIGRATION	-0.45	-1.95
GOBP_TUMOR_NECROSIS_FACTOR_SUPERFAMILY_CYTOKINE_PRODUCTION	-0.36	-1.86
GOBP_NEGATIVE_REGULATION_OF_SIGNALING	-0.19	-1.77
GOBP_REGULATION_OF_CATION_TRANSMEMBRANE_TRANSPORT	-0.32	-1.99
GOBP_POSITIVE_REGULATION_OF_TRANSMEMBRANE_RECEPTOR_ PROTEIN_SERINE_THREONINE_KINASE_SIGNALING_PATHWAY	-0.4	-1.99
GOBP_REGULATION_OF_LIPID_TRANSPORT	-0.37	-1.96
GOBP_MOLTING_CYCLE	-0.44	-1.95
GOBP_FATTY_ACID_TRANSPORT	-0.45	-1.94
GOBP_ACTOMYOSIN_STRUCTURE_ORGANIZATION	-0.36	-1.89
GOBP_CELL_CELL_JUNCTION_ASSEMBLY	-0.43	-1.89
GOBP_NEURAL_CREST_CELL_DIFFERENTIATION	-0.45	-1.88
GOBP_SENSORY_PERCEPTION_OF_PAIN	-0.45	-1.88
GOBP_ALPHA_BETA_T_CELL_ACTIVATION	-0.4	-1.88
GOBP_RESPONSE_TO_CAMP	-0.46	-1.87
GOBP_GRANULOCYTE_MIGRATION	-0.35	-1.87
GOBP_REGULATION_OF_CANONICAL_WNT_SIGNALING_PATHWAY	-0.32	-1.85
GOBP_SKIN_DEVELOPMENT	-0.28	-1.85
GOBP_ACTIN_FILAMENT_ORGANIZATION	-0.25	-1.85
GOBP_CHEMOKINE_PRODUCTION	-0.42	-1.82
GOBP_EMBRYO_DEVELOPMENT	-0.18	-1.69
GOBP_REGULATION_OF_INTRACELLULAR_SIGNAL_TRANSDUCTION	-0.16	-1.64
GOBP_POSITIVE_REGULATION_OF_CELL_SUBSTRATE_ADHESION	-0.42	-1.92
GOBP_WOUND_HEALING	-0.24	-1.89
GOBP_POSITIVE_REGULATION_OF_RESPONSE_TO_EXTERNAL_STIMULUS	-0.26	-1.88
GOBP_PLASMA_MEMBRANE_ORGANIZATION	-0.42	-2.01
GOBP_POSITIVE_REGULATION_OF_LIPID_LOCALIZATION	-0.46	-2.01
GOBP_PHOSPHATIDYLCHOLINE_METABOLIC_PROCESS	-0.49	-1.96
GOBP_MEMORY	-0.45	-1.96
GOBP_REGULATION_OF_REGULATED_SECRETORY_PATHWAY	-0.44	-1.94
GOBP_POSITIVE_REGULATION_OF_VASCULATURE_DEVELOPMENT	-0.36	-1.91
GOBP_NEGATIVE_REGULATION_OF_IMMUNE_SYSTEM_PROCESS	-0.27	-1.89
GOBP_DETECTION_OF_STIMULUS_INVOLVED_IN_SENSORY_PERCEPTION	-0.43	-1.85
GOBP_POSITIVE_REGULATION_OF_CYTOSKELETON_ORGANIZATION	-0.36	-1.84
GOBP_REGULATION_OF_ACTIN_FILAMENT_BASED_PROCESS	-0.28	-1.82

GOBP_POSITIVE_REGULATION_OF_EPITHELIAL_CELL_DIFFERENTIATION	-0.42	-1.82
GOBP_SYNAPTIC_SIGNALING	-0.24	-1.78
GOBP_DEVELOPMENTAL_GROWTH_INVOLVED_IN_MORPHOGENESIS	-0.3	-1.76
GOBP_CELLULAR_RESPONSE_TO_HORMONE_STIMULUS	-0.22	-1.69
GOBP_RESPONSE_TO_ORGANIC_CYCLIC_COMPOUND	-0.19	-1.68
GOBP_CELLULAR_RESPONSE_TO_ALCOHOL	-0.47	-1.97
GOBP_POSITIVE_REGULATION_OF_LEUKOCYTE_MEDIATED_IMMUNITY	-0.37	-1.87
GOBP_REGULATION_OF_CELL_MORPHOGENESIS_INVOLVED_IN_DIFFERENTIATION	-0.44	-1.82
GOBP_POSITIVE_REGULATION_OF_PROTEIN_KINASE_ACTIVITY	-0.23	-1.67
GOBP_REGULATION_OF_PROTEIN_KINASE_ACTIVITY	-0.21	-1.65
GOBP_POSITIVE_REGULATION_OF_NEURON_PROJECTION_DEVELOPMENT	-0.47	-1.93
GOBP_NEGATIVE_REGULATION_OF_CANONICAL_WNT_SIGNALING_PATHWAY	-0.41	-1.82
GOBP_ENDOTHELIUM_DEVELOPMENT	-0.37	-1.82
GOBP_STEM_CELL_DIFFERENTIATION	-0.3	-1.79
GOBP_DEVELOPMENTAL_MATURATION	-0.3	-1.79
GOBP_REGIONALIZATION	-0.26	-1.7
GOBP_OSTEOBLAST_DIFFERENTIATION	-0.33	-1.89
GOBP_REGULATION_OF_DEFENSE_RESPONSE	-0.22	-1.76
GOBP_CELLULAR_RESPONSE_TO_NITROGEN_COMPOUND	-0.2	-1.7
GOBP_SUPRAMOLECULAR_FIBER_ORGANIZATION	-0.2	-1.69
GOBP_MUSCLE_SYSTEM_PROCESS	-0.24	-1.68
GOBP_POSITIVE_REGULATION_OF_CELL_DEATH	-0.2	-1.67
GOBP_RESPONSE_TO_ABIOTIC_STIMULUS	-0.17	-1.62
GOBP_SKIN_EPIDERMIS_DEVELOPMENT	-0.44	-1.93
GOBP_REGULATION_OF_MUSCLE_CONTRACTION	-0.37	-1.89
GOBP_POSITIVE_REGULATION_OF_DNA_BINDING_TRANSCRIPTION_FACTOR_ACTIVITY	-0.29	-1.77
GOBP_NEGATIVE_REGULATION_OF_LEUKOCYTE_CELL_CELL_ADHESION	-0.35	-1.77
GOBP_DIGESTIVE_SYSTEM_PROCESS	-0.37	-1.74
GOBP_NEGATIVE_REGULATION_OF_HEMOPOIESIS	-0.38	-1.72
GOBP_POSITIVE_REGULATION_OF_CYTOKINE_PRODUCTION	-0.23	-1.67
GOBP_IMMUNE_SYSTEM_DEVELOPMENT	-0.17	-1.6
GOBP_POSITIVE_REGULATION_OF_LEUKOCYTE_PROLIFERATION	-0.35	-1.83
GOBP_EAR_DEVELOPMENT	-0.32	-1.76
GOBP_INTEGRIN_MEDIATED_SIGNALING_PATHWAY	-0.4	-1.8
GOBP_CARDIAC_MUSCLE_TISSUE_DEVELOPMENT	-0.31	-1.78
GOBP_REGULATION_OF_LYMPHOCYTE_MEDIATED_IMMUNITY	-0.35	-1.77
GOBP_REGULATION_OF_REPRODUCTIVE_PROCESS	-0.35	-1.73
GOBP_REGULATION_OF_CELLULAR_RESPONSE_TO_GROWTH_FACTOR_STIMULUS	-0.27	-1.66
GOBP_POSITIVE_REGULATION_OF_SUPRAMOLECULAR_FIBER_ORGANIZATION	-0.38	-1.78
GOBP_AMIDE_TRANSPORT	-0.28	-1.74
GOBP_MESENCHYME_DEVELOPMENT	-0.27	-1.68

GOBP_REGULATION_OF_CELL_MATRIX_ADHESION	-0.4	-1.79
GOBP_CELL_KILLING	-0.4	-1.72
GOBP_CAMERA_TYPE_EYE_MORPHOGENESIS	-0.43	-1.89
GOBP_LIPID_STORAGE	-0.43	-1.75
GOBP_CAMERA_TYPE_EYE_DEVELOPMENT	-0.26	-1.72
GOBP_NEUTROPHIL_CHEMOTAXIS	-0.35	-1.71
GOBP_REGULATION_OF_TRANS_SYNAPTIC_SIGNALING	-0.29	-1.82
GOBP_RENAL_SYSTEM_PROCESS	-0.4	-1.77
GOBP_MYELOID_LEUKOCYTE_MIGRATION	-0.28	-1.76
GOBP_NEGATIVE_REGULATION_OF_TRANSMEMBRANE_ RECEPTOR_PROTEIN_SERINE_THREONINE_KINASE_SIGNALING_PATHWAY	-0.39	-1.76
GOBP_POSITIVE_REGULATION_OF_GTPASE_ACTIVITY	-0.29	-1.75
GOBP_CARDIAC_CHAMBER_MORPHOGENESIS	-0.37	-1.8
GOBP_REGULATION_OF_SYNAPTIC_PLASTICITY	-0.41	-1.74
GOBP_MUSCLE_CELL_DIFFERENTIATION	-0.24	-1.72
GOBP_RESPONSE_TO_INSULIN	-0.3	-1.7
GOBP_AMINO_ACID_TRANSMEMBRANE_TRANSPORT	-0.41	-1.7
GOBP_LIPID_HOMEOSTASIS	-0.27	-1.69
GOBP_FATTY_ACID_METABOLIC_PROCESS	-0.22	-1.59
GOBP_MUCOPOLYSACCHARIDE_METABOLIC_PROCESS	-0.42	-1.8
GOBP_ENSHEATHMENT_OF_NEURONS	-0.39	-1.77
GOBP_CELL_ADHESION_MEDIATED_BY_INTEGRIN	-0.41	-1.71
GOBP_FATTY_ACID_DERIVATIVE_METABOLIC_PROCESS	-0.38	-1.69
GOBP_XENOBIOTIC_METABOLIC_PROCESS	-0.36	-1.65
GOBP_CELLULAR_ION_HOMEOSTASIS	-0.2	-1.6
GOBP_NEGATIVE_REGULATION_OF_LYMPHOCYTE_ACTIVATION	-0.36	-1.77
GOBP_GASTRULATION	-0.3	-1.72
GOBP_REGULATION_OF_BMP_SIGNALING_PATHWAY	-0.37	-1.7
GOBP_MESENCHYMAL_CELL_DIFFERENTIATION	-0.29	-1.77
GOBP_REGULATION_OF_ADAPTIVE_IMMUNE_RESPONSE	-0.35	-1.76
GOBP_RESPONSE_TO_STEROID_HORMONE	-0.25	-1.73
GOBP_DEMETHYLATION	-0.42	-1.7
GOBP_MUSCLE_CONTRACTION	-0.27	-1.7
GOBP_TERPENOID_METABOLIC_PROCESS	-0.38	-1.68
GOBP_NEURAL_PRECURSOR_CELL_PROLIFERATION	-0.32	-1.65
GOBP_METAL_ION_HOMEOSTASIS	-0.2	-1.63
GOBP_MUSCLE_TISSUE_DEVELOPMENT	-0.23	-1.61
GOBP_CARDIAC_CHAMBER_DEVELOPMENT	-0.32	-1.69
GOBP_MAINTENANCE_OF_LOCATION	-0.26	-1.65
GOBP_REGULATION_OF_CELLULAR_COMPONENT_SIZE	-0.24	-1.63
GOBP_POSITIVE_REGULATION_OF_CATALYTIC_ACTIVITY	-0.16	-1.52
GOBP_CELL_MATURATION	-0.34	-1.75

GOBP_BIOLOGICAL_PROCESS_INVOLVED_IN_SYMBIOTIC_INTERACTION	-0.27	-1.73
GOBP_REGULATION_OF_WNT_SIGNALING_PATHWAY	-0.27	-1.69
GOBP_REGULATION_OF_CELL_SIZE	-0.31	-1.68
GOBP_REGULATION_OF_PHAGOCYTOSIS	-0.35	-1.74
GOBP_REGULATION_OF_PEPTIDE_TRANSPORT	-0.35	-1.73
GOBP_SKELETAL_SYSTEM_MORPHOGENESIS	-0.3	-1.68
GOBP_TOLL_LIKE_RECEPTOR_SIGNALING_PATHWAY	-0.35	-1.64
GOBP_MUSCLE_STRUCTURE_DEVELOPMENT	-0.18	-1.5
GOBP_LIPID_METABOLIC_PROCESS	-0.14	-1.49
GOBP_MALE_SEX_DIFFERENTIATION	-0.35	-1.71
GOBP_BIOLOGICAL_PROCESS_INVOLVED_IN_INTERACTION_WITH_HOST	-0.32	-1.71
GOBP_GLOMERULUS_DEVELOPMENT	-0.41	-1.7
GOBP_CELLULAR_RESPONSE_TO_OXYGEN_LEVELS	-0.32	-1.69
GOBP_REGULATION_OF_PRODUCTION_OF_MOLECULAR_ MEDIATOR_OF_IMMUNE_RESPONSE	-0.35	-1.67
GOBP_CELLULAR_RESPONSE_TO_PEPTIDE	-0.24	-1.65
GOBP_REGULATION_OF_INFLAMMATORY_RESPONSE	-0.23	-1.64
GOBP_REGULATION_OF_VESICLE_MEDIATED_TRANSPORT	-0.21	-1.63
GOBP_RESPONSE_TO_KETONE	-0.31	-1.7
GOBP_BRANCHING_MORPHOGENESIS_OF_AN_EPITHELIAL_TUBE	-0.36	-1.69
GOBP_LYMPHOCYTE_MIGRATION	-0.4	-1.67
GOBP_NEGATIVE_REGULATION_OF_SUPRAMOLECULAR_FIBER_ORGANIZATION	-0.34	-1.65
GOBP_CARTILAGE_DEVELOPMENT	-0.34	-1.73
GOBP_REGULATION_OF_NEURON_DIFFERENTIATION	-0.32	-1.69
GOBP_RESPONSE_TO_GROWTH_FACTOR	-0.2	-1.6
GOBP_NEPHRON_DEVELOPMENT	-0.36	-1.73
GOBP_NEGATIVE_REGULATION_OF_ION_TRANSPORT	-0.36	-1.63
GOBP_BONE_DEVELOPMENT	-0.31	-1.59
GOBP_NOTCH_SIGNALING_PATHWAY	-0.32	-1.72
GOBP_REGULATED_EXOCYTOSIS	-0.3	-1.63
GOBP_REGULATION_OF_CELL_PROJECTION_ASSEMBLY	-0.32	-1.63
GOBP_REGULATION_OF_EPITHELIAL_CELL_DIFFERENTIATION	-0.31	-1.68
GOBP_POSITIVE_REGULATION_OF_JNK_CASCADE	-0.38	-1.65
GOBP_PEPTIDE_TRANSPORT	-0.3	-1.61
GOBP_ANATOMICAL_STRUCTURE_MATURATION	-0.28	-1.6
GOBP_POSITIVE_REGULATION_OF_INFLAMMATORY_RESPONSE	-0.32	-1.6
GOBP_POSITIVE_REGULATION_OF_DEFENSE_RESPONSE	-0.26	-1.67
GOBP_GRANULOCYTE_CHEMOTAXIS	-0.31	-1.62
GOBP_POSITIVE_REGULATION_OF_TRANSFERASE_ACTIVITY	-0.19	-1.52
GOBP_ION_HOMEOSTASIS	-0.18	-1.51
GOBP_REGULATION_OF_BODY_FLUID_LEVELS	-0.2	-1.49
GOBP_NEGATIVE_REGULATION_OF_WNT_SIGNALING_PATHWAY	-0.32	-1.7

GOBP_REGULATION_OF_NIK_NF_KAPPAB_SIGNALING	-0.41	-1.68
GOBP_PRIMARY_ALCOHOL_METABOLIC_PROCESS	-0.35	-1.64
GOBP_PEPTIDYL_TYROSINE_DEPHOSPHORYLATION	-0.37	-1.63
GOBP_ADENYLATE_CYCLASE_MODULATING_G_PROTEIN_ COUPLED_RECEPTOR_SIGNALING_PATHWAY	-0.34	-1.59
GOBP_NEGATIVE_REGULATION_OF_NEURON_PROJECTION_DEVELOPMENT	-0.37	-1.58
GOBP_REGULATION_OF_MUSCLE_SYSTEM_PROCESS	-0.3	-1.61
GOBP_REGULATION_OF_AXONOGENESIS	-0.33	-1.52
GOBP_LEUKOCYTE_CHEMOTAXIS	-0.27	-1.64
GOBP_MYOBLAST_DIFFERENTIATION	-0.37	-1.59
GOBP_NEGATIVE_REGULATION_OF_TRANSCRIPTION_BY_RNA_POLYMERASE_II	-0.17	-1.44
GOBP_JNK_CASCADE	-0.31	-1.63
GOBP_TEMPERATURE_HOMEOSTASIS	-0.33	-1.61
GOBP_MONONUCLEAR_CELL_MIGRATION	-0.3	-1.6
GOBP_ALPHA_BETA_T_CELL_DIFFERENTIATION	-0.37	-1.56
GOBP_RESPONSE_TO_ACID_CHEMICAL	-0.32	-1.55
GOBP_POSITIVE_REGULATION_OF_STRESS_ACTIVATED _PROTEIN_KINASE_SIGNALING_CASCADE	-0.32	-1.54
GOBP_EXOCYTOSIS	-0.22	-1.52
GOBP_LOCOMOTORY_BEHAVIOR	-0.34	-1.57
GOBP_RESPONSE_TO_TRANSFORMING_GROWTH_FACTOR_BETA	-0.25	-1.52
GOBP_POSITIVE_REGULATION_OF_LYMPHOCYTE_MEDIATED_IMMUNITY	-0.35	-1.63
GOBP_PHOSPHOLIPID_TRANSPORT	-0.35	-1.58
GOBP_CALCIUM_ION_TRANSMEMBRANE_TRANSPORT	-0.28	-1.55
GOBP_REGULATION_OF_PROTEIN_SECRETION	-0.26	-1.52
GOBP_TUBE_FORMATION	-0.33	-1.59
GOBP_PLACENTA_DEVELOPMENT	-0.28	-1.53
GOBP_LEARNING	-0.37	-1.58
GOBP_RESPONSE_TO_TUMOR_NECROSIS_FACTOR	-0.28	-1.61
GOBP_REGULATION_OF_LIPID_METABOLIC_PROCESS	-0.22	-1.5
GOBP_REGULATION_OF_LEUKOCYTE_MIGRATION	-0.26	-1.59
GOBP_RECEPTOR_SIGNALING_PATHWAY_VIA_STAT	-0.35	-1.54
GOBP_RESPONSE_TO_CYTOKINE	-0.16	-1.44
GOBP_AMINOGLYCAN_METABOLIC_PROCESS	-0.31	-1.57
GOBP_CELLULAR_RESPONSE_TO_INSULIN_STIMULUS	-0.28	-1.51
GOBP_PROTEIN_LOCALIZATION_TO_EXTRACELLULAR_REGION	-0.24	-1.52
GOBP_NEUROTRANSMITTER_TRANSPORT	-0.29	-1.5
GOBP_POSITIVE_REGULATION_OF_MOLECULAR_FUNCTION	-0.14	-1.38
GOBP_ZYMOGEN_ACTIVATION	-0.36	-1.57
GOBP_NEURON_MIGRATION	-0.32	-1.53
GOBP_FOREBRAIN_DEVELOPMENT	-0.22	-1.49
GOBP_REGULATION_OF_PROTEIN_SERINE_THREONINE_KINASE_ACTIVITY	-0.22	-1.5
GOBP_RESPONSE_TO_PURINE_CONTAINING_COMPOUND	-0.32	-1.52

GOBP_RECEPTOR_MEDIATED_ENDOCYTOSIS	-0.28	-1.51
GOBP_TISSUE_REGENERATION	-0.38	-1.58
GOBP_CYTOKINE_MEDIATED_SIGNALING_PATHWAY	-0.22	-1.5
GOBP_REPRODUCTIVE_SYSTEM_DEVELOPMENT	-0.2	-1.45
GOBP_LIPID_CATABOLIC_PROCESS	-0.23	-1.45
GOBP_CELLULAR_RESPONSE_TO_ORGANIC_CYCLIC_COMPOUND	-0.19	-1.44
GOBP_REGULATION_OF_ACTIN_FILAMENT_ORGANIZATION	-0.27	-1.54
GOBP_MYELOID_CELL_DIFFERENTIATION	-0.2	-1.45
GOBP_DIGESTIVE_SYSTEM_DEVELOPMENT	-0.3	-1.53
GOBP_CARBOHYDRATE_HOMEOSTASIS	-0.28	-1.52
GOBP_GLAND_DEVELOPMENT	-0.21	-1.47
GOBP_DETECTION_OF_ABIOTIC_STIMULUS	-0.3	-1.47
GOBP_NEGATIVE_REGULATION_OF_BINDING	-0.35	-1.53
GOBP_THIOESTER_METABOLIC_PROCESS	-0.32	-1.49
GOBP_REGULATION_OF_PEPTIDASE_ACTIVITY	-0.2	-1.44
GOBP_MONOVALENT_INORGANIC_CATION_HOMEOSTASIS	-0.3	-1.55
GOBP_RESPONSE_TO_INORGANIC_SUBSTANCE	-0.18	-1.43
GOBP_REGULATION_OF_RESPONSE_TO_STRESS	-0.14	-1.37
GOBP_PROGRAMMED_CELL_DEATH	-0.13	-1.33
GOBP_REGULATION_OF_MUSCLE_CELL_DIFFERENTIATION	-0.32	-1.55
GOBP_ANTIGEN_RECEPTOR_MEDIATED_SIGNALING_PATHWAY	-0.27	-1.45
GOBP_PRODUCTION_OF_MOLECULAR_MEDIATOR_ INVOLVED_IN_INFLAMMATORY_RESPONSE	-0.36	-1.48
GOBP_RESPONSE_TO_METAL_ION	-0.21	-1.47
GOBP_RESPONSE_TO_PEPTIDE_HORMONE	-0.21	-1.48
GOBP_REGULATION_OF_CELL_DEATH	-0.13	-1.34
GOBP_REGULATION_OF_SMALL_GTPASE_MEDIATED_SIGNAL_TRANSDUCTION	-0.24	-1.49
GOBP_NEUROMUSCULAR_PROCESS	-0.34	-1.47
GOBP_NEURAL_TUBE_DEVELOPMENT	-0.29	-1.53
GOBP_MAINTENANCE_OF_PROTEIN_LOCATION	-0.35	-1.48
GOBP_CONNECTIVE_TISSUE_DEVELOPMENT	-0.24	-1.45
GOBP_CELL_PROJECTION_ASSEMBLY	-0.2	-1.41
GOBP_REGULATION_OF_STRESS_ACTIVATED_PROTEIN_KINASE_SIGNALING_CASCADE	-0.27	-1.53
GOBP_NEUTRAL_LIPID_METABOLIC_PROCESS	-0.31	-1.46
GOBP_MORPHOGENESIS_OF_EMBRYONIC_EPITHELIUM	-0.31	-1.46
GOBP_NIK_NF_KAPPAB_SIGNALING	-0.34	-1.49
GOBP_REGULATION_OF_B_CELL_ACTIVATION	-0.36	-1.48
GOBP_KERATINOCYTE_DIFFERENTIATION	-0.29	-1.46
GOBP_EPIDERMAL_CELL_DIFFERENTIATION	-0.25	-1.45
GOBP_POSITIVE_REGULATION_OF_GENE_EXPRESSION	-0.14	-1.34
GOBP_CILIUM_MOVEMENT	-0.35	-1.44
GOBP_TRANSFORMING_GROWTH_FACTOR_BETA_RECEPTOR_SIGNALING_PATHWAY	-0.26	-1.41

GOBP_REGULATION_OF_SUPRAMOLECULAR_FIBER_ORGANIZATION	-0.21	-1.4
GOBP_POSITIVE_REGULATION_OF_RESPONSE_TO_BIOTIC_STIMULUS	-0.26	-1.42
GOBP_REGULATION_OF_FATTY_ACID_METABOLIC_PROCESS	-0.36	-1.46
GOBP_PROTEIN_AUTOPHOSPHORYLATION	-0.26	-1.46
GOBP_REGULATION_OF_CELL_SHAPE	-0.31	-1.46
GOBP_POSITIVE_REGULATION_OF_WNT_SIGNALING_PATHWAY	-0.31	-1.44
GOBP_LEUKOCYTE_DEGRANULATION	-0.32	-1.43
GOBP_POSITIVE_REGULATION_OF_PROTEIN_SERINE_THREONINE _KINASE_ACTIVITY	-0.24	-1.4
GOBP_NEGATIVE_REGULATION_OF_PHOSPHORUS_METABOLIC_PROCESS	-0.19	-1.38
GOBP_CARDIAC_VENTRICLE_MORPHOGENESIS	-0.35	-1.44
GOBP_CYTOSKELETON_ORGANIZATION	-0.13	-1.31
GOBP_REGULATION_OF_TRANSFERASE_ACTIVITY	-0.15	-1.33
GOBP_CELL_RECOGNITION	-0.29	-1.42
GOBP_HEMOSTASIS	-0.21	-1.4
GOBP_SMOOTH_MUSCLE_CONTRACTION	-0.31	-1.44
GOBP_ICOSANOID_METABOLIC_PROCESS	-0.31	-1.43
GOBP_POSITIVE_REGULATION_OF_CELL_PROJECTION_ORGANIZATION	-0.25	-1.37
GOBP_POSITIVE_REGULATION_OF_PROTEIN_SECRETION	-0.28	-1.39
GOBP_CELLULAR_LIPID_METABOLIC_PROCESS	-0.14	-1.31
GOBP_REGULATION_OF_LEUKOCYTE_CHEMOTAXIS	-0.29	-1.42
GOBP_DIVALENT_INORGANIC_CATION_HOMEOSTASIS	-0.19	-1.34
GOBP_REGULATION_OF_NEUROTRANSMITTER_LEVELS	-0.27	-1.42
GOBP_PRODUCTION_OF_MOLECULAR_MEDIATOR_OF_IMMUNE_RESPONSE	-0.24	-1.36
GOBP_POSITIVE_REGULATION_OF_NF_KAPPAB_TRANSCRIPTION_ FACTOR_ACTIVITY	-0.28	-1.36
GOBP_REGULATION_OF_RESPONSE_TO_OXIDATIVE_STRESS	-0.31	-1.4
GOBP_VESICLE_MEDIATED_TRANSPORT_IN_SYNAPSE	-0.28	-1.36
GOBP_AXON_EXTENSION	-0.31	-1.42
GOBP_MULTI_ORGANISM_REPRODUCTIVE_PROCESS	-0.14	-1.31
GOBP_MECHANORECEPTOR_DIFFERENTIATION	-0.34	-1.36
GOBP_SENSORY_PERCEPTION_OF_MECHANICAL_STIMULUS	-0.26	-1.37
GOBP_REGULATION_OF_DNA_BINDING_TRANSCRIPTION_FACTOR_ACTIVITY	-0.2	-1.37
GOBP_REGULATION_OF_RESPONSE_TO_BIOTIC_STIMULUS	-0.2	-1.35
GOBP_REGULATION_OF_EPITHELIAL_CELL_APOPTOTIC_PROCESS	-0.32	-1.41
GOBP_CELLULAR_RESPONSE_TO_PEPTIDE_HORMONE_STIMULUS	-0.24	-1.4
GOBP_CELL_ACTIVATION_INVOLVED_IN_IMMUNE_RESPONSE	-0.22	-1.38
GOBP_POSITIVE_REGULATION_OF_CALCIUM_ION_TRANSPORT	-0.32	-1.36
GOBP_NEGATIVE_REGULATION_OF_CELL_PROJECTION_ORGANIZATION	-0.28	-1.36
GOBP_DEVELOPMENT_OF_PRIMARY_SEXUAL_CHARACTERISTICS	-0.24	-1.36
GOBP_ACUTE_INFLAMMATORY_RESPONSE	-0.29	-1.39
GOBP_PATTERN_RECOGNITION_RECEPTOR_SIGNALING_PATHWAY	-0.25	-1.34
GOBP_MAINTENANCE_OF_LOCATION_IN_CELL	-0.24	-1.32

GOBP_MESODERM_DEVELOPMENT	-0.28	-1.34
GOBP_SEX_DIFFERENTIATION	-0.22	-1.34
GOBP_RETINA_DEVELOPMENT_IN_CAMERA_TYPE_EYE	-0.27	-1.35
GOBP_REGULATION_OF_AUTOPHAGY	-0.21	-1.34
GOBP_MYELOID_CELL_ACTIVATION_INVOLVED_IN_IMMUNE_RESPONSE	-0.29	-1.33
GOBP_REGULATION_OF_PHOSPHOPROTEIN_PHOSPHATASE_ACTIVITY	-0.32	-1.36
GOBP_RESPONSE_TO_TEMPERATURE_STIMULUS	-0.28	-1.35
GOBP_MUSCLE_CELL_DEVELOPMENT	-0.28	-1.39
GOBP_TELENCEPHALON_DEVELOPMENT	-0.25	-1.37
GOBP_RESPONSE_TO_OXIDATIVE_STRESS	-0.18	-1.3
GOBP_POSITIVE_REGULATION_OF_TUMOR_NECROSIS_FACTOR _SUPERFAMILY_CYTOKINE_PRODUCTION	-0.33	-1.4
GOBP_REGULATION_OF_REACTIVE_OXYGEN_SPECIES_METABOLIC_PROCESS	-0.25	-1.36
GOBP_AGING	-0.23	-1.33
GOBP_EMBRYONIC_SKELETAL_SYSTEM_DEVELOPMENT	-0.29	-1.35
GOBP_CELLULAR_COMPONENT_ASSEMBLY_INVOLVED_IN_MORPHOGENESIS	-0.34	-1.41
GOBP_POSITIVE_REGULATION_OF_PRODUCTION_OF_MOLECULAR _MEDIATOR_OF_IMMUNE_RESPONSE	-0.31	-1.33
GOBP_SKELETAL_MUSCLE_ORGAN_DEVELOPMENT	-0.29	-1.34
GOBP_DEVELOPMENTAL_PROCESS_INVOLVED_IN_REPRODUCTION	-0.14	-1.25
GOBP_REGULATION_OF_CYTOSKELETON_ORGANIZATION	-0.17	-1.31
GOBP_ADAPTIVE_THERMOGENESIS	-0.27	-1.31
GOBP_RESPONSE_TO_NITROGEN_COMPOUND	-0.13	-1.26
GOBP_STRESS_ACTIVATED_PROTEIN_KINASE_SIGNALING_CASCADE	-0.22	-1.34
GOBP_PEPTIDYL_AMINO_ACID_MODIFICATION	-0.13	-1.26
GOBP_SMAD_PROTEIN_SIGNAL_TRANSDUCTION	-0.32	-1.32
GOBP_REGULATION_OF_RAS_PROTEIN_SIGNAL_TRANSDUCTION	-0.26	-1.32
GOBP_POSITIVE_REGULATION_OF_PROTEIN_LOCALIZATION_TO_CELL_PERIPHERY	-0.32	-1.32
GOBP_NUCLEOSIDE_BISPHOSPHATE_METABOLIC_PROCESS	-0.25	-1.32
GOBP_REGULATION_OF_CELLULAR_KETONE_METABOLIC_PROCESS	-0.27	-1.31
GOBP_EPITHELIAL_TUBE_FORMATION	-0.28	-1.32
GOBP_ISOPRENOID_METABOLIC_PROCESS	-0.25	-1.31
GOBP_ENDOCRINE_SYSTEM_DEVELOPMENT	-0.28	-1.3
GOBP_POSITIVE_REGULATION_OF_LEUKOCYTE_MIGRATION	-0.26	-1.31
GOBP_NEGATIVE_REGULATION_OF_DEVELOPMENTAL_GROWTH	-0.28	-1.3
GOBP_NEURAL_TUBE_FORMATION	-0.29	-1.27
GOBP_FATTY_ACID_BIOSYNTHETIC_PROCESS	-0.25	-1.31
GOBP_VIRAL_LIFE_CYCLE	-0.18	-1.27
GOBP_REGULATION_OF_ACTIN_FILAMENT_LENGTH	-0.26	-1.3
GOBP_NEGATIVE_REGULATION_OF_VIRAL_PROCESS	-0.32	-1.3
GOBP_POSITIVE_REGULATION_OF_INNATE_IMMUNE_RESPONSE	-0.26	-1.28
GOBP_REGULATION_OF_MYELOID_CELL_DIFFERENTIATION	-0.22	-1.28
GOBP_VENTRICULAR_SEPTUM_DEVELOPMENT	-0.32	-1.3

**GOBP_REGULATION_OF_CARBOHYDRATE_METABOLIC_PROCESS**	**-0.24**	**-1.28**
**GOBP_REGULATION_OF_CALCIUM_ION_TRANSPORT**	**-0.24**	**-1.29**
**GOBP_NEGATIVE_REGULATION_OF_NEURON_APOPTOTIC_PROCESS**	**-0.27**	**-1.28**
**GOBP_REGULATION_OF_PROTEIN_BINDING**	**-0.24**	**-1.28**
**GOBP_OLEFINIC_COMPOUND_METABOLIC_PROCESS**	**-0.28**	**-1.26**
